# Threshold driven contagion on weighted networks

**DOI:** 10.1038/s41598-018-21261-9

**Published:** 2018-02-15

**Authors:** Samuel Unicomb, Gerardo Iñiguez, Márton Karsai

**Affiliations:** 1 Univ Lyon, ENS de Lyon, Inria, CNRS, UCB Lyon 1, LIP UMR 5668, IXXI, F-69342 Lyon, France; 2 Instituto de Investigaciones en Matemáticas Aplicadas y en Sistemas, Universidad Nacional Autónoma de México, 04510 Ciudad de México, Mexico; 3 Department of Computer Science, Aalto University School of Science, P.O. Box 15500, Espoo, Finland

## Abstract

Weighted networks capture the structure of complex systems where interaction strength is meaningful. This information is essential to a large number of processes, such as threshold dynamics, where link weights reflect the amount of influence that neighbours have in determining a node's behaviour. Despite describing numerous cascading phenomena, such as neural firing or social contagion, the modelling of threshold dynamics on weighted networks has been largely overlooked. We fill this gap by studying a dynamical threshold model over synthetic and real weighted networks with numerical and analytical tools. We show that the time of cascade emergence depends non-monotonously on weight heterogeneities, which accelerate or decelerate the dynamics, and lead to non-trivial parameter spaces for various networks and weight distributions. Our methodology applies to arbitrary binary state processes and link properties, and may prove instrumental in understanding the role of edge heterogeneities in various natural and social phenomena.

## Introduction

Weighted networks provide meaningful representations of the architecture of a large number of complex systems where interacting entities, represented as nodes in a graph, are connected with links weighted by the strength of their interactions. Weighted networks are ubiquitous in biological^1^, ecological^2^, infrastructure^3–5^, social^6–9^, information, and economic^10,11^ systems, just to mention a few. Their analysis has been in focus from the early stages of complex networks research^12,13^, with several measures^14–16^ and models^17,18^ introduced. These studies show that link weights in real networks are usually heterogeneous, may be correlated with the network structure^6,19^, and can even capture signed relationships^20^. More importantly, weights help to differentiate links of varying importance, influence, and role. On a microscopic level, weights identify the most relevant neighbours of a node^21^; on a network level, they indicate links with special roles or positions in the system^6,19^. Such information is crucial for dynamical processes evolving on weighted networks. Examples can be found in epidemiology, where important ties maintained by frequent interactions may enhance the spread of infection locally, while ties with infrequent interactions but located between densely connected parts of the network may suppress diffusion globally^19,22^. Link weights are also relevant in phenomena like random walks, spin models, synchronisation, evolutionary games, as well as cascading failures. Despite this, weighted networks have been less studied than their unweighted counterparts, especially for threshold driven processes, which play an essential role in systems of self-organised criticality^23–25^, epidemiology^26^, firing neurons^27–29^, or social contagion^6,30^.

In threshold driven processes, the state of an entity changes when the concentration of incoming stimuli or cumulating force reaches a certain threshold. Some typical examples are neural systems^27,28^, earthquakes^31^, and solar flares^32,33^, commonly identified as self-organised critical systems driven by integrate-and-fire mechanisms. Thresholds play a role in some epidemic diseases, such as tuberculosis and dysentery^26^, where infection requires the concentration of pathogens in an individual to overcome a threshold. Moreover, thresholds are associated with social contagion phenomena, where social influence from acquaintances may change the behaviour of an individual after reaching a cognitive limit. Studies of so-called *complex contagion* date back to Schelling, Axelrod, and Granovetter, but have recently attracted interest thanks to the seminal cascade model by Watts^30^, and also to the enormous amount of digital data on human behaviour collected to observe, analyse and model social contagion. In threshold models on networks links are usually considered unweighted, such that the stimuli or influence arriving from each neighbour contributes equally to reaching the behavioural threshold. Although this assumption simplifies their modelling, it does not lead to an accurate representation of real world dynamics. For example, in neural systems synaptic connections have weights that quantify the strength of incoming stimuli, and contribute unequally in bringing neurons to an excited state, as recognised recently in models of neural population dynamics^34^. In social systems link weights are associated with tie strengths that quantify the social influence that individuals have on their peers. Measurement of tie strength is a long standing challenge, but it is generally accepted that social ties are not equal, as some are more influential than others on one’s decision making. Surprisingly, apart from some recent studies^35–37^, weights have been commonly overlooked in models of threshold driven phenomena.

Our aim is to close this gap by exploring the effect of weight heterogeneities on threshold driven contagion processes. We first study a dynamical variant of the Watts cascade model on a simple system, a random regular network with a bimodal weight distribution. We then provide an analytical solution of the dynamics, for arbitrary degrees and weights, together with numerical simulations and combinatorial arguments to show that the speed of spreading depends non-monotonously on the extent of weight heterogeneity and may drastically accelerate or decelerate as compared to the unweighted case, even for fixed thresholds. We also observe this effect under more realistic synthetic scenarios, such as scale-free networks and lognormal weight distributions, as well as in data-driven simulations over large-scale empirical weighted networks. Our contribution is a meaningful step forward in the largely unexplored modelling of dynamical processes with heterogeneous interactions, typical in neural systems and social contagion. Moreover, our results may have broader implications as our methodology is not specific to threshold dynamics and may be easily extended to any binary state process, while our study and conclusions may be useful in accurately modelling other dynamical phenomena over weighted networks.

## Results

### Threshold model and approximate solutions

To study threshold driven dynamical processes over weighted networks we build on the seminal model proposed by Watts^30^. Following its standard formulation^30,38–40^, we define a monotone binary-state dynamics over a weighted, undirected network of size *N*. Degrees take discrete values *k* = 0, …, *N* − 1 according to a distribution *P*(*k*), and edge weights *w* > 0 are continuous variables with distribution *P*(*w*). The edge weight *w*_*ij*_ represents the capacity of connected nodes *i* and *j* to influence each other. Accordingly, the node strength ${q}_{k}(i)={\sum }_{j=1}^{k}{w}_{ij}$ is the total influence node *i* receives from its *k* neighbours. Like in other conventional models of spreading dynamics^41^, nodes can be in two mutually exclusive states, susceptible (initially all nodes), or infected (also called adopter in the social contagion literature). A susceptible node can become infected either spontaneously with rate *p*^39,40^, or if the influence of its infected neighbours exceeds a given threshold *ϕ* (0 < *ϕ* < 1). However, influence may vary from neighbour to neighbour. We implement this idea by defining the partial strength ${q}_{m}(i)={\sum }_{j=1}^{m}{w}_{ij}$ associated with the influence of the *m* infected neighbours on node *i* (where 0 ≤ *m* ≤ *k*). If the condition *q*_*m*_ ≥ *ϕq*_*k*_ is fulfilled, node *i* becomes infected and remains so indefinitely. For simplicity we assume that all nodes have the same threshold *ϕ*, just as in many other studies^30,38^ (implementation details in Methods).

**Figure 1:**
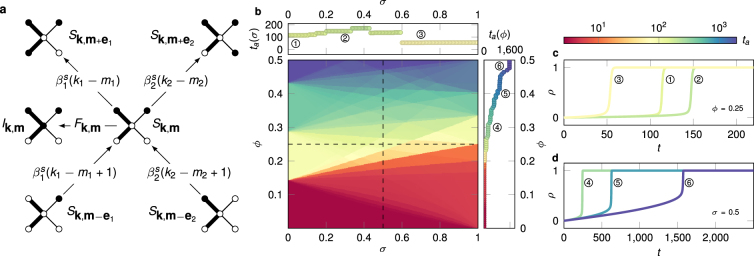
Threshold driven contagion and cascade evolution on weighted networks. (**a**) Transitions into and out of a class *S*_**k**,**m**_ of susceptible nodes in a network with two weights (*n* = 2). Susceptible nodes may enter or leave *S*_**k**,**m**_ with rate ${\beta }_{1}^{s}$, ${\beta }_{2}^{s}$ via the infection of neighbours with weight type *j* = 1,2, or via their own infection with rate *F*_**k**,**m**_. (**b**) Parameter dependence of the time *t*_*a*_ of cascade emergence (main panel) on a random regular network with degree *k* = 7, and bimodal weight distribution with mean *μ* = 1 and standard deviation *σ* (for further details see text). Cascade speed is measured by the time *t*_*a*_ to reach 75% infection. For fixed threshold *ϕ* and varying *σ*, *t*_*a*_ changes non-monotonously, while for fixed *σ* and varying *ϕ*, dynamics slows down for increasing *ϕ* (top/right panels, corresponding to horizontal/vertical dashed lines in main panel). (**c**,**d**) Spreading time series *ρ*(*t*) for selected parameter values in (**b**). Curves in panel (c) were measured with fixed *ϕ* = 0.25 at *σ* = 0 (curve 1), 0.3 (2), and 0.7 (3), while curves in panel (d) were measured with fixed *σ* = 0.5 at *ϕ* = 0.3 (4), 0.4 (5), and 0.5 (6). Simulation results in (**b**–**d**) are averages of 25 simulations with *p* = 2 × 10^−4^ and *N* = 10^4^.

We explore this model analytically by extending Gleeson’s approximate master equation (AME) formalism for stochastic binary-state dynamics^41–44^ over weighted networks. Although we only consider monotone dynamics in detail, our formalism can easily be extended to arbitrary binary state processes (see Supplementary Information [SI]). The original AME formalism considers unweighted networks with an arbitrary degree distribution, which are otherwise maximally random. It assumes that all nodes with degree *k* and number of infected neighbours *m* follow the same dynamics, forming a node class (*k*, *m*) that can be described by a single pair of rate equations. In order to extend this formalism to weighted networks, we discretise *P*(*w*) and assume only *n* possible weight types *w*_*j*_, such that all distinct weights in the network are contained in the weight vector **w** = (*w*_1_, …, *w*_*n*_)^T^. Then, a node in class (k, m) has *k*_*j*_ links with weight *w*_*j*_ and *m*_*j*_ = 0, …, *k*_*j*_ infected neighbours across these links, such that ${\rm{k}}={\sum }_{j=1}^{n}{k}_{j}$ and $m={\sum }_{j=1}^{n}{m}_{j}$. Furthermore, we can define a degree vector **k** = (*k*_1_, …, *k*_*n*_)^T^ and a partial degree vector **m** = (*m*_1_, …, *m*_*n*_)^T^, generalising the strength and partial strength to *q*_***k***_ = **k** ⋅ **w** and *q*_**m**_ = **m** ⋅ **w**, respectively. Nodes in class (**k**, **m**) have identical strengths and partial strengths, and follow the same pair of rate equations for the fraction *s*_**k**,**m**_(*t*) [*i*_**k**,**m**_(*t*)] of **k**-nodes that are susceptible (infected) at time *t* and have partial degree vector **m** (see Methods and SI). Fig. 1a illustrates the case of *n* = 2, and shows the possible transitions into and out of the class of susceptible nodes with degree vector **k** = (2, 2) and partial degree vector **m** = (1, 1).

In threshold driven contagion a susceptible node can become infected in two ways, either spontaneously with rate *p*, or if its weighted threshold *ϕ* is reached. As such, the infection rate of susceptible nodes in class (**k**, **m**) is

1 $${F}_{{\bf{k}},{\bf{m}}}=\{\begin{array}{cc}p & \quad {q}_{{\bf{m}}} < \varphi {q}_{{\bf{k}}}\\ 1 & \quad {q}_{{\bf{m}}}\ge \varphi {q}_{{\bf{k}}}\end{array},\quad k > 0,$$

with *F*_**0**,**0**_ = *p*. The stepwise nature of *F*_**k**,**m**_ allows us to map the rate equations for *s*_**k**,**m**_ and *i*_**k**,**m**_ to a reduced-dimension system, as has been done previously for the Watts threshold model^41,42,44^ and unweighted complex contagion^39,40^. Namely, if we consider as aggregated variables the density *ρ*(*t*) of infected nodes and the probability *ν*_*j*_(*t*) that a randomly chosen neighbour (across a *j*-type edge) of a susceptible node is infected (for definitions see Methods), then the description of the dynamics can be reduced to the system of *n* + 1 equations

2a $${\dot{\nu }}_{j}={g}_{j}({\boldsymbol{\nu }},t)-{\nu }_{j},$$

2b $$\dot{\rho }=h({\boldsymbol{\nu }},t)-\rho ,$$

where ***ν*** = (*ν*_1_, …, *ν*_*n*_)^T^ is the vector of probabilities *ν*_*j*_ for all weight types, and *g*_*j*_(***ν***, *t*) and *h*(***ν***, *t*) are functions of binomial terms (see Methods and SI).

### Regular networks with bimodal weights

To study the dynamics of our model we first consider a simple structure, the configuration-model *k*-regular network, with *k* = 7. Edge weights are sampled from a bimodal distribution with *n* = 2 values, denoted strong (*w*_1_) and weak (*w*_2_). The weight distribution is characterised by its average *μ*, standard deviation *σ* ≥ 0, and the fraction *δ* of links that are strong. Thus, weights take the values ${w}_{1}=\mu +\sigma \sqrt{\mathrm{(1}-\delta )/\delta }$ and ${w}_{2}=\mu -\sigma \sqrt{\delta /\mathrm{(1}-\delta )}$. The parameter *δ* contributes to the skewness of *P*(*w*), initially fixed to the symmetric case *δ* = 0.5. The parameter *σ* interpolates weight heterogeneity between the homogeneous case of an unweighted network (*σ* = 0), and the most heterogeneous case of a diluted network ($\sigma =\mu \sqrt{\mathrm{(1}-\delta )/\delta }$), where only strong links have influence and the weak are functionally absent. After fixing the spontaneous infection rate *p* and skewness *δ*, our model has only two parameters, *σ* and *ϕ* (Fig. 1b). Similar to other dynamical cascade models^39,40^, contagion initially evolves at a linear rate until the density *ρ*(*t*) of infected nodes reaches a critical value, triggering a rapid cascade of infection that spreads through the whole network (sample scenarios in Fig. 1c,d). Thus, to characterise the speed of dynamics we introduce the quantity *t*_*a*_, the time when infection density reaches a set value (*ρ* = 0.75), called the absolute time of cascade emergence. We measure *t*_*a*_ via numerical simulations of the (*σ*, *ϕ*)-parameter space (Fig. 1b), which shows unexpected dependencies on both parameters. On one hand, for fixed *σ* and increasing *ϕ* the dynamics slows down, since nodes with higher thresholds require more infected neighbours to become infected. On the other, for fixed *ϕ* the dynamics depends *non-monotonously* on *σ*, where cascades may evolve either faster or slower as we increase weight heterogeneity, relative to the unweighted case (*σ* = 0). Note that similar behaviour is exhibited after setting *p* = 0 and introducing an initial seed, although in the following we use the dynamic variant of the Watts model, where *p* > 0.

**Figure 2:**
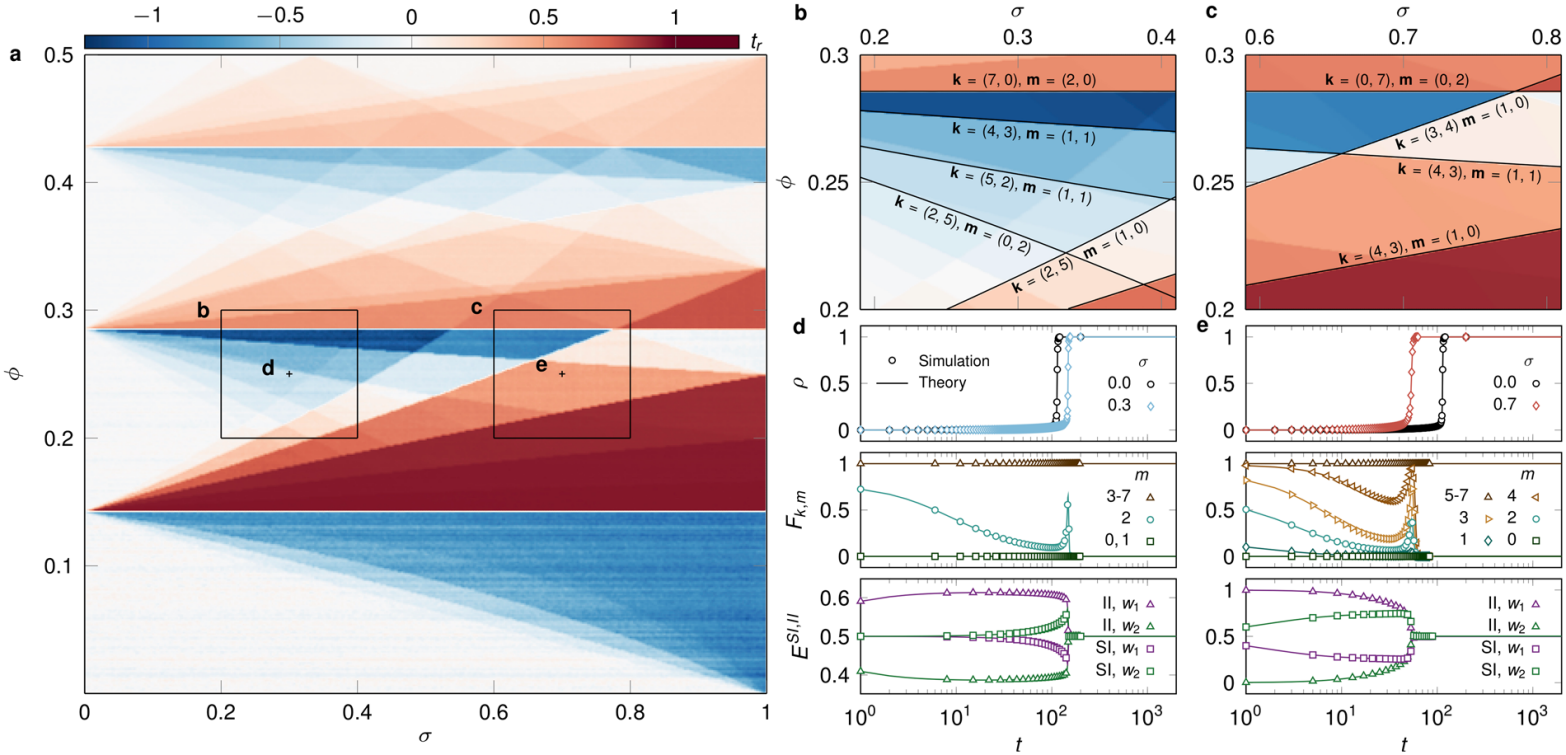
Relative time of threshold driven cascades on weighted networks. (**a**) Relative time *t*_*r*_ of cascade emergence on (*σ*, *ϕ*)-parameter space, simulated over *k*-regular regular networks (*k* = 7) with *μ* = 1, *δ* = 0.5, *p* = 2 × 10^−4^, *N* = 10^4^ and averaged over 25 realisations. Time of cascades for given *ϕ* is either higher or lower than the corresponding case (0, *ϕ*) of an unweighted network. (**b**,**c**) Selected regions of parameter space in (**a**), where *t*_*r*_ is instead calculated from the numerical solution of the AME systems in Eq. 2. Boundaries are obtained from a combinatorial argument (see Methods and SI) for various (**k**, **m**) classes. For example, the boundary **k** = (2, 5), **m** = (1, 0) separates networks where nodes with *k*_1_ = 2 strong links and *k*_2_ = 5 weak links may (or may not) be infected by *m*_1_ = 1 strong infected neighbour. (**d**,**e**) Quantities characterising the dynamics in simulations (symbols) and AMEs (lines) for *ϕ* = 0.25 and *σ* corresponding to the unweighted case, as well as to a slow (**d**) or fast (**e**) cascade. Quantities are the infection density *ρ*(*t*) (upper panel), aggregated infection rate *F*_*k*,*m*_(*t*) for various numbers of infected neighbours *m* (middle panel), and fractions of strong (*w*_1_) and weak (*w*_2_) links inside the infected cluster [*E*^*II*^(*t*)] and on the surface of it [*E*^*SI*^(*t*)] (bottom panel). Simulation and theory results in (**a**–**e**) agree perfectly.

We concentrate on the *σ* dependency by calculating ${t}_{r}=[{t}_{a}\mathrm{(0},\varphi )-{t}_{a}(\sigma ,\varphi )]/{t}_{a}\mathrm{(0},\varphi )$, the time of cascade emergence relative to the unweighted case with the same *ϕ* value. (Fig. 2a). The relative time *t*_*r*_ will be positive if the weighted process evolves faster than the unweighted case, zero if they evolve at the same speed, and negative if slower than the unweighted case. The (*σ*, *ϕ*)-parameter space for *t*_*r*_ is highly structured and driven by competing effects of key (*k*,*m*) classes, which either reduce or enhance the speed of the spreading process as compared to the unweighted case. We also explore the corresponding numerical solution of the AME systems in Eq. 2, as well as an independent combinatorial solution for the boundaries between regions of low and high cascade speed (Fig. 2b,c) (see Methods and SI). Both the AME and combinatorial solutions perfectly recover the parameter space obtained by simulations. To further explore how weight heterogeneities produce slow or fast cascades, we partition the system according to the number *m* of infected neighbours required for infection, and measure the aggregated infection rate ${F}_{k,m}(t)={\sum }_{{\bf{k}},{\bf{m}}}P({\bf{k}}){F}_{{\bf{k}},{\bf{m}}}{s}_{{\bf{k}},{\bf{m}}}(t)/{\sum }_{{\bf{k}},{\bf{m}}}P({\bf{k}}){s}_{{\bf{k}},{\bf{m}}}(t)$ and other determinant quantities in several spreading scenarios (Fig. 2d,e).

In the neutral scenario, all (**k**, **m**) classes of the weighted network share the same dynamics as the corresponding (*k*, *m*) class in an unweighted network, so *F*_*k*,*m*_ = *p* or 1 and weights have no impact on contagion, meaning *t*_*r*_ = 0. In a decelerative scenario like *ϕ* = 0.25 and *σ* = 0.3 (Fig. 2d), *F*_*k*,*m*_ for any *m* is equal to its unweighted counterpart, except for the *m* = 2 class. Here, the adoption rate is 1 in the unweighted case but strongly suppressed in the weighted case, thus decreasing the overall spreading speed. For an accelerative scenario, like *ϕ* = 0.25 and *σ* = 0.7, competing effects from several (**k**, **m**) classes combine to determine the overall dynamics (Fig. 2e). The rate *F*_*k*,*m*_ for *m* = 2, …, 4 is lower than 1 which is a decelerative effect (as in the previous case), but the rate *F*_*k*,1_, which is equal to *p* in the unweighted case, is significantly larger than *p* here. Since at the early stages of contagion the number of nodes in class *m* = 1 is larger than in any other class with *m* > 1, spreading evolves rapidly to an early cascade. It should be noted that competition between the accelerative and decelerative effects of the weight distribution is one of the defining characteristics of threshold driven contagion on weighted networks. It is this competition that leads to the interference patterns evident in Fig. 2a.

Furthermore, an asymmetry is observed to emerge in the fractions of weak and strong links connecting infected [*E*^*II*^(*t*)] or susceptible and infected [*E*^*SI*^(*t*)] nodes (see Methods). Since strong ties contribute the most to reaching the threshold of a node, they participate earlier in the contagion and comprise most ties in the infected subgraph. Conversely, weak ties dominate the surface of the cascade by connecting infected and susceptible nodes. This asymmetry in edge types is an essential feature of weighted contagion that is trivially absent in the unweighted case. This asymmetry appears in cases of both accelerated and decelerated spreading, with amplitude dependent on the absolute value of the relative time of contagion. Note that results from simulations (symbols in Fig. 2d and e) and AMEs (lines in Fig. 2d and e) agree very well, for all quantities studied.

**Figure 3:**
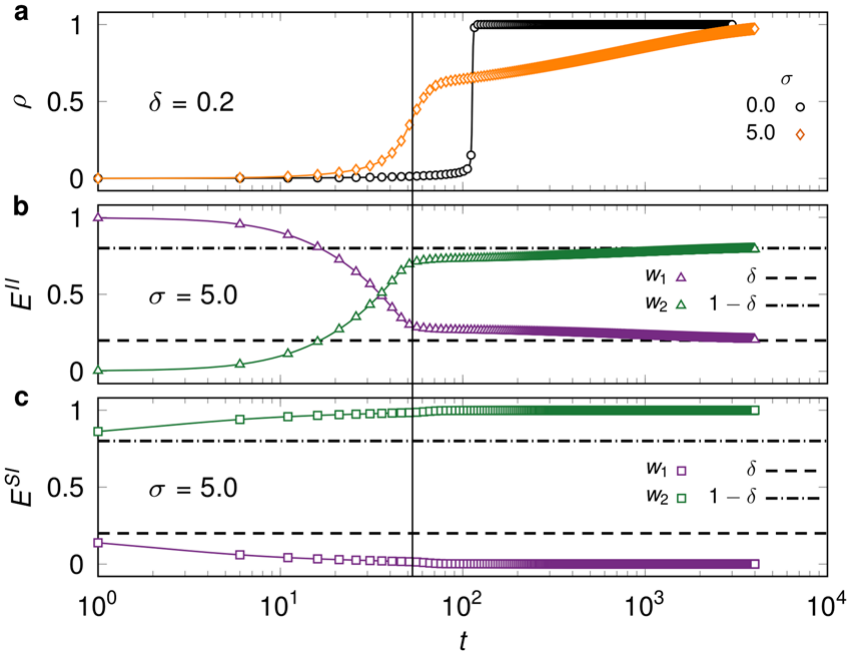
Effect of skewed weight distributions on cascade evolution. (**a**) Infection density *ρ*(*t*) on *k*-regular networks (*k* = 7) and a bimodal weight distribution with *μ* = 3 and *δ* = 0.2, both for unweighted (*σ* = 0) and heterogeneous (*σ* > 0) cases. (**b**,**c**) Fractions of strong (*w*_1_) and weak (*w*_2_) links connecting two infected nodes in the bulk of the infected component [*E*^*II*^ (*t*), b] and susceptible and infected nodes on its surface [*E*^*SI*^(*t*), c] in the heterogeneous spreading scenario of (a). Simulations (symbols) are averaged over 25 realisations with *p* = 2 × 10^−4^ and *N* = 10^4^, and compared with the corresponding AME solution of Eqs (3)-(2) (lines). Dashed lines are the expected fractions of weak and strong links as determined by *δ*, and the vertical line shows the inflection point of *ρ* in the heterogeneous case of (**a**), which coincides with a turning point of *E*^*II*^ in (**b**).

Up until now we have considered the symmetric case *δ* = 0.5 with equal numbers of strong and weak links. However, by skewing the weight distribution we observe an additional effect of weight heterogeneities on the spreading behaviour. When *δ* = 0.2 the extent of the cascade decreases for large *σ* with respect to the unweighted case (Fig. 3a). In this case, despite their sparsity, strong links again drive the contagion, but are soon exhausted causing spreading to slow down and continue via spontaneous or infrequent threshold driven infections over weak ties (Fig. 3b). Indeed, strong links dominate the bulk of the infected component, but disappear quickly from its surface (Fig. 3c). These so-called *partial cascades*, which do not infect the whole system through the cascade, are associated with skewness and a sufficiently large standard deviation in the weight distribution and are reminiscent of the slow spreading caused by immune nodes, as well as low connectivity networks in unweighted complex contagion^30,39,40^. Overall, we identify non-monotonous spreading behaviour and partial cascades as the main consequences of weight heterogeneities in threshold driven contagion.

### Heterogeneous synthetic and real networks

**Figure 4:**
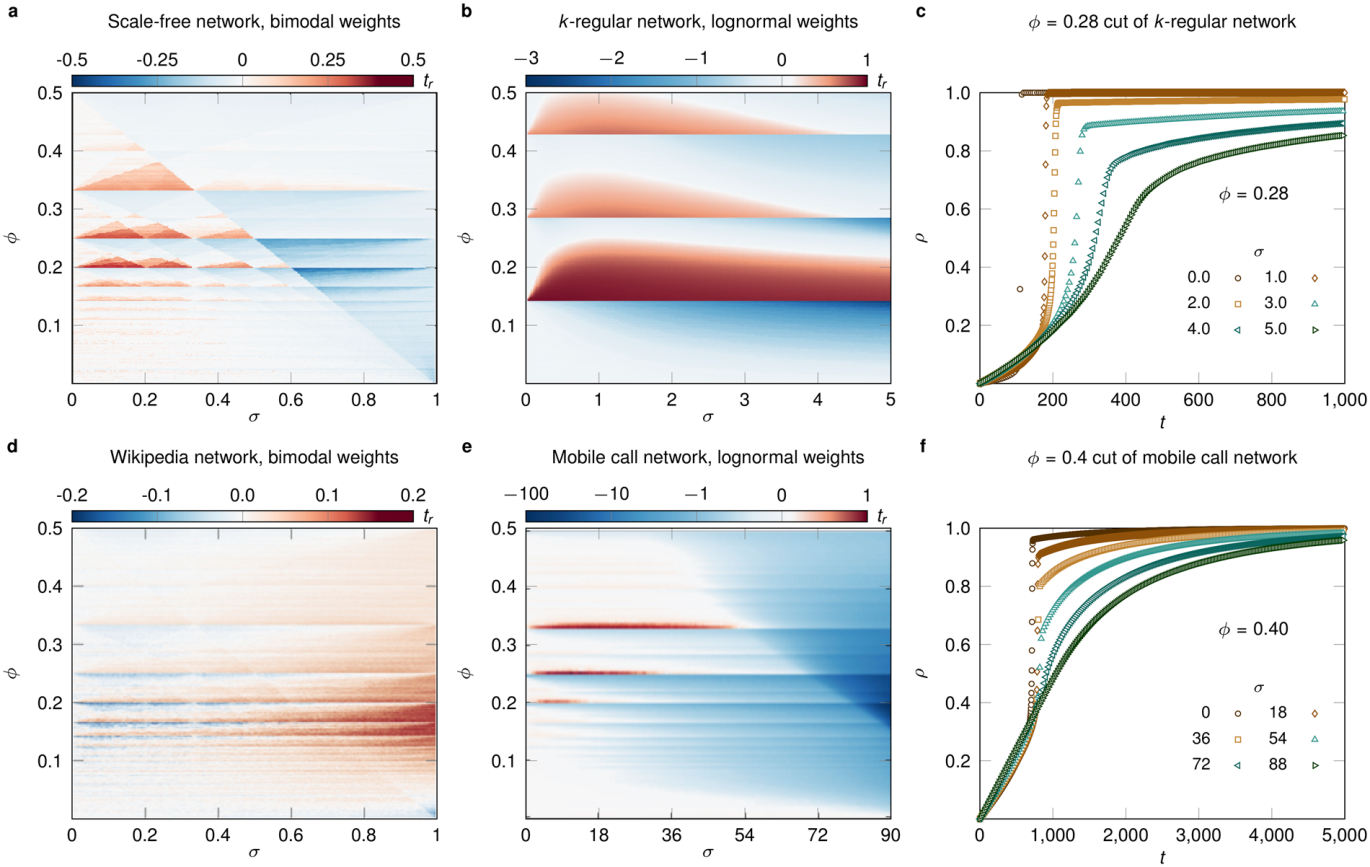
Threshold contagion on heterogeneous synthetic and real networks. (**a**) Relative time *t*_*r*_ of cascade emergence on (*σ*, *ϕ*)-parameter space, simulated over synthetic scale-free networks with degree exponent *τ* = 2.5, average degree *z* = 4.54 and minimum degree *k*_min_ = 2. Link weights are bimodally distributed with *μ* = 1 and *δ* = 0.5. (**b**) Same as (a) but over a *k*-regular network (*k* = 7) and a lognormal weight distribution with *μ* = 1. (**c**) Infection density *ρ*(*t*) in the lognormal case of (**b**) for *ϕ* = 0.28 and varying *σ*. The skewness of the weight distribution induces partial cascades in contagion. (**d**) Relative time *t*_*r*_ of cascade emergence on (*σ*, *ϕ*)-parameter space, simulated over a signed social network of Wikipedia editors with heterogeneous degrees and skewed bimodal weight distribution (see Methods). (**e**) Same as (**d**) but over a mobile phone call (MPC) network with heterogeneously distributed degrees and weights, and *μ* = 37.7. (**f**) Infection density *ρ*(*t*) in the MPC network of (**e**) for *ϕ* = 0.4 and varying *σ*. Synthetic networks in (**a,b**) have *N* = 10^4^ and parameter spaces are averaged over 25 realisations. Parameter space in (**d**) is averaged over 10^3^ realisations, while (**e**) is the result of a single realisation. All simulations correspond to *p* = 2 × 10^−4^.

Although regular networks and bimodal weights are useful in characterising the qualitative impact of weights on contagion, they are rather unrealistic since real complex networks commonly appear with broad degree and weight distributions^3^. Thus, in the following we explore how threshold driven contagion is influenced by weights using simulations in heterogeneous synthetic and real weighted networks (Fig. 4). We expect degree heterogeneities to affect threshold driven processes since thresholds are defined relative to the degree (or strength) of nodes. As a first step we take synthetic scale-free networks from the configuration model with degree distribution $P(k)\sim {k}^{-\tau }$ and exponent *τ* = 2.5, but maintain a bimodal weight distribution with *μ* = 1 and *δ* = 0.5 as in the previous section (Fig. 4a). The increased number of (**k**, **m**) classes (due to degree heterogeneity) fragments the (*σ*, *ϕ*)-parameter space for *t*_*r*_, but its structure still reveals areas of slow and fast cascades and can be explained by the same arguments used for the *k*-regular case. Real world examples of this synthetic structure are signed social networks, like the network of Wikipedia editors^45^, where degrees are broadly distributed and edge signs indicate the parity (or binary weight) of a social interaction like trust, intimacy, or influence. We also simulate our threshold model over the Wikipedia social network by associating + and − tie signs with strong (*w*_1_) and weak (*w*_2_) links, thus obtaining a weighted network with *δ* = 0.88 and arbitrary *σ* (Fig. 4d) (see Methods). Despite complex structural correlations potentially present in the real data, the Wikipedia (*σ*, *ϕ*)-parameter space is qualitatively similar to the case of a synthetic scale-free degree distribution, although these correlations and the high *δ* value transform the areas of relative acceleration and deceleration. To further validate these observations, we have also analysed configuration-model random networks and another empirical signed network, the Pardus dataset^20^ (see SI).

As with degree heterogeneities, weights in empirical networks are broadly distributed and approximated by scale-free or lognormal distributions, which we address by exploring the threshold model on *k*-regular networks (*k* = 7) and a lognormal weight distribution with average *μ* = 1 (Fig. 4b). Even though all nodes have the same degree, diversity of weight values increases the number of (**k**, **m**) classes, smoothing out the (*σ*, *ϕ*)-parameter space with respect to the bimodal case but qualitatively maintaining its non-monotonous patterns of slow and fast cascades. The standard deviation *σ* controls the skewness of the weight distribution and determines the temporal evolution of contagion, promoting partial cascades for large *σ* (Fig. 4c). Finally, we consider threshold driven contagion in a large empirical weighted social network, an aggregated mobile phone call (MPC) network, where weights are proportional to the number of calls between individuals (Fig. 4e) (see Methods). This network has broad degree and weight distributions^9^, communities, degree correlations and Granovetter-type degree-weight correlations^46^. Despite this added complexity, the (*σ*, *ϕ*)-parameter space of the MPC network is qualitatively similar to previous cases, apart from the magnitude of the decelerative effect when weights are strongly heterogeneous. As before, skewness in the weight distribution temporally inhibits contagion and induces partial cascades (Fig. 4f). Our data-driven simulations show that, even in empirical networks of vastly different origins, threshold driven contagion strongly depends on link weights via simple mechanisms that can be understood by master equations or combinatorial arguments. This dependence may be responsible for the diverse dynamical scenarios of threshold driven contagion observed in nature, like the diffusion of information in techno-social networks, which typically reaches a limited population, but can occasionally unfold globally through slow or fast cascades of adoption.

## Discussion

In complex networks, weights quantify the strength of interactions between nodes and distinguish neighbours by their relevance or influence. Threshold driven contagion in empirical settings is particularly sensitive to link weights, since influence between connected nodes may vary enormously, thus changing the temporal pattern of global spreading. Examples of real-world threshold driven processes over weighted networks can be found in epidemiology, where contagion is enabled by direct human interactions that occur at varying frequencies throughout the population^26^. In the case of social contagion, like the spreading of information, service or product adoption, participation in collective movements, or the adoption of behavioural patterns, heterogeneities in social tie strengths are relevant as they may reflect the strength of influence of people on each other^7,19,22,46^. In neural systems, the synaptic weight (a function of several variables) may vary heterogeneously between connected neurons and even in time due to synaptic plasticity^34,47^. Despite this broad set of real-world examples, threshold driven contagion has mostly only been studied over unweighted networks where links are considered equal.

Our aim in this paper has been to address this shortfall by systematically studying a threshold model on synthetic and empirical weighted networks. We explore networks with increasing complexity, from configuration-model networks with bimodal or lognormal weights, to real world networks with broad degree and weight distributions as well as multiple correlations. We show that threshold driven contagion depends non-monotonously on weight heterogeneity, creating slow or fast cascades relative to the equivalent unweighted spreading process. Via numerical simulations, master equations and combinatorial arguments, we find that this effect is the result of competing configurations of degree, weight, and infected neighbours that slow down or speed up contagion. We also observe that an imbalance in the amount of large and small weights leads to partial cascades, and smoother temporal patterns of spreading than those in unweighted networks. By analysing a range of degree and weight configurations, we show that these features are systemic and thus may drive a variety of real world contagion phenomena.

Our contribution opens up directions of research in the largely unexplored area of dynamical processes with heterogeneous interactions. First, the weight-based, master equation formalism described here can be modified to consider any interaction quality like direction and type, thus providing analytical tools to characterise threshold driven contagion in temporal and multiplex networks^48,49^. The master equation formalism also provides means of studying the cascade-size phase space^30^, where one might expect a non-monotonic dependence of cascade size on weight heterogeneity, an effect observed in ref.^50^ due to threshold heterogeneity. In addition, although the effects of initial seed size on the emerging cascades have been addressed earlier^51^, it has never been studied on weighted structures, and may prove an interesting direction to explore. Second, our methodology may be used to describe any binary-state dynamics and thus a broad class of empirical processes over weighted networks. We expect our results to find meaningful applications in fields where threshold driven contagion is relevant, like computational epidemiology, neural networks, and social contagion. In these fields our modelling framework, which distinguishes the varied roles and influence of links, may lead to advances in the understanding and prediction of specific temporal features of global pandemics, collective neural firing, or the adoption of innovations and behavioural norms.

## Methods

### Numerical implementation

We implement weighted complex contagion numerically via Monte Carlo simulations of a monotone binary-state dynamics. Node states change from susceptible to infected in asynchronous random order in a series of time steps. Once a node state changes from susceptible to infected, it remains so for the rest of the dynamics, thus ensuring a frozen final state for the finite system where no more state changes take place. Each time step consists of *N* node updates. In each node update, a randomly selected node becomes spontaneously infected with probability *p*, or else it adopts only if the weighted threshold rule *q*_**m**_ ≥ *ϕq*_**k**_ is satisfied (see Eq. 1). This is the case if the selected node is susceptible; if the node is infected, no action is taken. We assume that nodes with *k* = 0 receive no influence from the rest of the network (for any value of *ϕ*), and therefore can only change state spontaneously. Regarding synthetic networks, we only consider configuration-model networks^52^ with an uncorrelated distribution of edge weights on top of them, i.e. an ensemble of networks specified by the distributions *P*(*k*) and *P*(*w*), but otherwise maximally random. Thus, the distributions *P*(*k*) and *P*(*w*) (together with *p*) determine the average topological state and dynamical evolution of the system.

### AMEs in weighted networks

The dynamics of our threshold model evolves in small time intervals *dt*. Accordingly, the rate equations for the fractions *s*_**k**,**m**_(*t*) [*i*_**k**,**m**_(*t*)] of **k**-nodes that are susceptible (infected) at time *t* and have partial degree vector ***m*** are

3a $$\frac{d}{dt}{s}_{{\bf{k}},{\bf{m}}}=-{F}_{{\bf{k}},{\bf{m}}}{s}_{{\bf{k}},{\bf{m}}}-\sum _{j=1}^{n}{\beta }_{j}^{s}({k}_{j}-{m}_{j}){s}_{{\bf{k}},{\bf{m}}}+\sum _{j=1}^{n}{\beta }_{j}^{s}({k}_{j}-{m}_{j}+\mathrm{1)}{s}_{{\bf{k}},{\bf{m}}-{{\bf{e}}}_{j}},$$

3b $$\frac{d}{dt}{i}_{{\bf{k}},{\bf{m}}}=+{F}_{{\bf{k}},{\bf{m}}}{s}_{{\bf{k}},{\bf{m}}}-\sum _{j=1}^{n}{\beta }_{j}^{i}({k}_{j}-{m}_{j}){i}_{{\bf{k}},{\bf{m}}}+\sum _{j=1}^{n}{\beta }_{j}^{i}({k}_{j}-{m}_{j}+\mathrm{1)}{i}_{{\bf{k}},{\bf{m}}-{{\bf{e}}}_{j}},$$

where *F*_**k**,**m**_ is the rate of infection of susceptible nodes in class (**k**, **m**), and the other terms quantify the rates at which susceptible nodes leave and enter the class (**k**, **m**) via the infection of susceptible neighbours. The *j*-th basis vector of dimension *n* is denoted by ***e***_*j*_ (${s}_{{\bf{k}},-{{\bf{e}}}_{j}}\equiv 0$), while ${\beta }_{j}^{s}(t)$ [${\beta }_{j}^{i}(t)$] is the rate at which a *j*-type susceptible neighbour of a susceptible (infected) node becomes infected (see Fig. 1a). The AME system (2) applies to all monotone binary-state dynamics over edge-heterogeneous networks, regardless of the form of *F*_**k**,**m**_, and its solution provides a very accurate description of the dynamics, even if the number of equations to solve grows rapidly with *n*. Moreover, variables in Eq. (3) satisfy the normalisation condition

4 $$\sum _{{\bf{m}}}{i}_{{\bf{k}},{\bf{m}}}+\sum _{{\bf{m}}}{s}_{{\bf{k}},{\bf{m}}}=1.$$

If **k** is distributed according to *P*(**k**), the probability that a randomly selected node has degree *k* and degree vector **k** is *P*(*k*)*P*(**k**). Then, the rates ${\beta }_{j}^{s}(t)$ and ${\beta }_{j}^{i}(t)$ are

5a $${\beta }_{j}^{s}(t)=\frac{\sum _{k,{\bf{k}},{\bf{m}}}\,P(k)P({\bf{k}})({k}_{j}-{m}_{j}){F}_{{\bf{k}},{\bf{m}}}{s}_{{\bf{k}},{\bf{m}}}(t)}{\sum _{k,{\bf{k}},{\bf{m}}}\,P(k)P({\bf{k}})({k}_{j}-{m}_{j}){s}_{{\bf{k}},{\bf{m}}}(t)}$$

and

5b $${\beta }_{j}^{i}(t)=\frac{\sum _{k,{\bf{k}},{\bf{m}}}\,P(k)P({\bf{k}})({k}_{j}-{m}_{j}){F}_{{\bf{k}},{\bf{m}}}{i}_{{\bf{k}},{\bf{m}}}(t)}{\sum _{k,{\bf{k}},{\bf{m}}}\,P(k)P({\bf{k}})({k}_{j}-{m}_{j}){i}_{{\bf{k}},{\bf{m}}}(t)},$$

where the sum over all degrees, strength and partial strength vectors is written explicitly as

6 $$\sum _{k,{\bf{k}},{\bf{m}}}=\sum _{k={k}_{{\rm{\min }}}}^{{k}_{{\rm{\max }}}}\sum _{{\bf{k}}}\sum _{{m}_{1}=0}^{{k}_{1}}\ldots \sum _{{m}_{n}=0}^{{k}_{n}}\mathrm{.}$$

The second sum runs over all strength vectors ***k*** = (*k*_1_, …, *k*_*n*_)^T^ satisfying the constraint $k={\sum }_{j=1}^{n}{k}_{j}$.

### Aggregated variables and the reduced AMEs

Variables in Eq. (2) are the fraction of infected nodes in the system,

7 $$\rho (t)=1-\sum _{k,{\bf{k}},{\bf{m}}}P(k)P({\bf{k}}){s}_{{\bf{k}},{\bf{m}}}(t),$$

and the probability that a randomly chosen neighbour (across a *j*-type edge) of a susceptible node is infected,

8 $${\nu }_{j}(t)=\sum _{k,{\bf{k}}}\,P(k)P({\bf{k}})\frac{\sum _{{\bf{m}}}{m}_{j}{s}_{{\bf{k}},{\bf{m}}}(t)}{\sum _{{\bf{m}}}{k}_{j}{s}_{{\bf{k}},{\bf{m}}}(t)}.$$

Also, Eq. (2) includes the functions of binomial terms

9a $${g}_{j}({\boldsymbol{\nu }},t)={f}_{t}+\mathrm{(1}-{f}_{t})\,\sum _{k,{\bf{k}}}\frac{{k}_{j}}{{z}_{j}}\,P(k)P({\bf{k}})\sum _{{q}_{{\bf{m}}}\ge \varphi {q}_{{\bf{k}}}}{B}_{{k}_{j}-1,{m}_{j}}({\nu }_{j})\prod _{i\ne j}^{n}{B}_{{k}_{i},{m}_{i}}({\nu }_{i}),$$

9b $$h({\boldsymbol{\nu }},t)={f}_{t}+\mathrm{(1}-{f}_{t})\,\sum _{k,{\bf{k}}}\,P(k)P({\bf{k}})\sum _{{q}_{{\bf{m}}}\ge \varphi {q}_{{\bf{k}}}}\prod _{j=1}^{n}{B}_{{k}_{j},{m}_{j}}({\nu }_{j}),$$

with *f*_*t*_ = 1−(1 − *p*)*e*^−*pt*^, *z*_*j*_ the average number of *j*-type edges of a node, and ${B}_{{k}_{j},{m}_{j}}=(\genfrac{}{}{0ex}{}{{k}_{j}}{{m}_{j}}){\rho }^{{m}_{j}}{(1-\rho )}^{{k}_{j}-{m}_{j}}$ the binomial distribution.

### Initial conditions

We assume that at time *t* = 0 there is an infinitesimally small fraction of infected nodes randomly distributed in the network, so the initial condition for Eq. (3) is

10 $${s}_{{\bf{k}},{\bf{m}}}\mathrm{(0)}=\prod _{j=1}^{n}{B}_{{k}_{j},{m}_{j}}\mathrm{(0).}$$

In the reduced AMEs, Eq. (10) corresponds to [***ν***(0), *ρ*(0)] = (**0**, **0**).

### Combinatorial solution of parameter space boundaries

Taking the equality in the threshold rule, Eq. 1, and writing *q*_**k**_, *q*_**m**_ explicitly, we obtain *ϕ* = **m** ⋅ **w**/**k** ⋅ **w**, where **w** implicitly depends on *σ*. After solving this equation for a given **k** and **m**, we associate the solution with a boundary line of *t*_*r*_ values in (*σ*,*ϕ*)-parameter space (Fig. 2b,c). These boundaries separate network configurations where the corresponding (**k**, **m**) class does or does not satisfy the threshold rule. For example, the boundary **k** = (3, 4), **m** = (1,0) (Fig. 2c) separates networks where nodes with *k*_1_ = 3 strong links and *k*_2_ = 4 weak links may be infected by only *m*_1_ = 1 strong infected neighbour. If two networks differ only in the rate of infection of nodes in this (**k**, **m**) class (so that one is eligible for infection and not the other), we observe a difference in spreading time (for details see SI).

### Bulk and interface of the contagion cluster

We characterise the effect of weights in threshold driven contagion by measuring how many *j*-type links, 1 ≤ *j* ≤ *n*, are on the bulk and at the surface of cascades. Explicitly, we compute the fraction of *j*-type links per node, connecting two infected nodes (the cascade bulk),

11 $${E}_{j}^{II}(t)=\frac{\sum _{k,{\bf{k}}{\boldsymbol{,}}{\bf{m}}}\,P(k)P(k){m}_{j}{i}_{{\bf{k}},{\bf{m}}}(t)}{\sum _{k,{\bf{k}}{\boldsymbol{,}}{\bf{m}}}\,P(k)P(k)m{i}_{{\bf{k}},{\bf{m}}}(t)},$$

and susceptible and infected nodes (cascade surface),

12 $${E}_{j}^{SI}(t)=\frac{\sum _{k,{\bf{k}}{\boldsymbol{,}}{\bf{m}}}\,P(k)P(k){m}_{j}{s}_{{\bf{k}},{\bf{m}}}(t)}{\sum _{k,{\bf{k}}{\boldsymbol{,}}{\bf{m}}}\,P(k)P(k)m{s}_{{\bf{k}},{\bf{m}}}(t)},$$

such that ∑_*j*_*E*^*II*^ = ∑_*j*_*E*^*SI*^ = 1. Now if *P*(*w*) is bimodal (*n* = 2), then ${E}_{1}^{SI}+{E}_{2}^{SI}=1$, ${E}_{1}^{II}+{E}_{2}^{II}=1$, and we may remove the index *j* (Figs 2d,e and 3c). The quantities ${E}_{j}^{II}$ and ${E}_{j}^{SI}$ diverge from 1/2 with amplitude dependent on the absolute difference of the speed from the dynamics on unweighted networks (*σ* = 0) where ${E}_{j}^{II}={E}_{j}^{SI}=1/2$.

### Data description

We perform data-driven simulations of our threshold model in two large-scale, empirical social networks. The first is a network of *N* = 138,592 English Wikipedia editors contributing to articles about politics. Each of the 740,397 directed links (defining an edit, revert, restore, or vote action in an article) has a sign (±), interpreted as the parity of trust between connected editors (for free access online and details see ref.^45^). In our study we remove self-loops and assume bidirectional links appear as undirected links with their original sign (if they shared the same sign), while choosing a sign randomly in the case where they appear with different signs (such edges only form 0.96% of the network, so their effect is not significant). Unidirectional links are also regarded as undirected with their original sign. Finally, we associate + and − tie signs to strong (*w*_1_) and weak (*w*_2_) links. The network has a broad degree distribution, a fraction *δ* = 0.88 of strong links and average weight *μ* = 2.7.

The second data set is an aggregated, static social network of *N* = 6,243,322 individuals connected by 16,783,865 undirected links with weights defined as the number of phone calls between people in an observation period of 6 months (a link exists if people have mutually called each other at least once). All individuals are customers of a single phone provider with 20% market share in an undisclosed European country. Degree and weight distributions are broad and can be approximated by power-law and lognormal distributions, respectively (for details see ref.^9^). Since for the MPC network *P*(*w*) is fixed, we introduce a method to scale *σ* without changing the shape of the distribution, described as follows. We first assume that the MPC network has a weight set *W* = {*w*_1_, …, *w*_|*E*|_}, where *w*_*i*_ is the weight of the *i*-th edge, and |*E*| is the number of edges in the network. This set has mean and variance

13 $$\mu =\frac{1}{|E|}\sum _{i=1}^{|E|}{w}_{i}\quad {\rm{and}}\quad {\sigma }^{2}=\frac{1}{|E|}\sum _{i=1}^{|E|}{({w}_{i}-\mu )}^{2}.$$

Now we consider a new weight set *W*′ = {*μ* + *α*(*w*_1_ − *μ*), …, *μ* + *α*(*w*_|*E*__|_ − *μ*)}, where we have applied the transformation ${w}_{i}^{\prime} =\mu +\alpha ({w}_{i}-\mu )$, *i* = 1, …, |*E*|, and 0 ≤* α* ≤ 1 is a tuning parameter. The limits of this transformation give a Dirac delta distribution (*α* = 0) or *P*(*w*) (*α* = 1). Substituting ${w}_{i}^{\prime}$ into the expression for *σ*, we see that the mean and standard deviation of the transformed weight set are *μ*′ = *μ* and *σ*′ *=* *ασ*. Then, we may obtain a new weight distribution retaining the shape of *P*(*w*) by applying the transformation ${w}_{i}\mapsto {w}_{i}^{\prime}$. If *σ*′ is the desired standard deviation, the required tuning parameter is *α* = *σ*′/*σ*.

### Data availability statement

The Wikipedia network data we used in Fig. 4d is freely available online at^45^. The mobile call data used in Fig. 4e and f are available from an undisclosed mobile operator but restrictions apply to the availability of these data, which were used under license for the current study, and so are not publicly available. Data may however be available from the authors upon reasonable request and with permission of the provider.

## Electronic supplementary material

Supplementary Information
