# Supplementary material for: Threshold driven contagion on weighted networks
Source: Sci Rep. 2018 Feb 15;8:3094. doi: 10.1038/s41598-018-21261-9 (PMC5814462; doi:10.1038/s41598-018-21261-9)
Supplement: Supplementary file 1 — Supplementary Information [file 41598_2018_21261_MOESM1_ESM.pdf]

Supplementary Information

Threshold driven contagion on weighted networks

S. Unicomb, G. Iñiguez, M. Karsai*

*Corresponding author email: marton.karsai@ens-lyon.fr

Contents

S1 Approximate master equations on weighted networks **2**

S1.1 Binary-state dynamics 2

S1.2 Monotone dynamics 4

S1.3 Monotone dynamics for bimodal weight distribution 5

S1.4 Reduced AMEs 7

S2 Combinatorial solution of parameter space boundaries **11**

S3 Comparison of numerical experiment and AME solutions **12**

S4 Other heterogeneous synthetic and real networks **13**

S1 Approximate master equations on weighted networks

In this section we justify and outline the derivation of approximate master equations (AMEs) for stochastic binary-state dynamics on weighted networks. We begin with a general derivation, and later show how this framework simplifies for monotone dynamics. We identify edge types by the value of their weights, however the formalism remains unchanged by distinguishing edge types with other link properties, such as direction or color. The following derivation builds upon a formalism developed by Gleeson [1–4].

S1.1 Binary-state dynamics

The theoretical framework of the AMEs applies to stochastic binary-state dynamics on random networks, where nodes have degree k with distribution $P(k)$. The network is assumed to be infinite and maximally random, meaning there is no correlation between k and any other graph property. We attribute to each node in the network one of two possible states, susceptible (S) or infected (I). We denote by m the number of infected neighbours of a node, with $0 \leq m \leq k$. We refer to m interchangeably as the infected neighbour count or partial degree. A susceptible node of degree k that has m infected neighbours belongs to the set $S_{k,m}$, and an infected node to the set $I_{k,m}$. As such the network can be partitioned into a finite number of sets, assuming $P(k)$ is bounded by a minimum and maximum degree $k_{\min} \leq k \leq k_{\max}$.

We may further partition $S_{k,m}$ and $I_{k,m}$ by assuming a finite number of edge types within the network, distinguished by their weight. If edge weights take one of n distinct values, it is instructive to introduce a weight vector $\mathbf{w} = (w_1, \ldots, w_n)^T$ to store the n values w_j. This could be generalized to any n-dimensional edge property vector. Further, we define the degree vector $\mathbf{k} = (k_1, \ldots, k_n)^T$ and partial degree vector $\mathbf{m} = (m_1, \ldots, m_n)^T$. Here, k_j and m_j respectively denote the number of neighbours and the number of infected neighbours of a node that are connected by an edge of weight w_j. These quantities are connected to the degree and partial degree via $k = \sum_j k_j$ and $m = \sum_j m_j$. Moreover, $S_{\mathbf{k},\mathbf{m}}$ and $I_{\mathbf{k},\mathbf{m}}$ denote the set of susceptible and infected nodes, respectively, that have degree vector $\mathbf{k}$ and partial degree vector $\mathbf{m}$. Every node in $S_{\mathbf{k},\mathbf{m}}$ belongs to a corresponding set $S_{k,m}$, as is the case for $I_{\mathbf{k},\mathbf{m}}$ and $I_{k,m}$. Finally, the size of these sets is quantified through $s_{\mathbf{k},\mathbf{m}}(t)$ and $i_{\mathbf{k},\mathbf{m}}(t)$, the fraction of nodes with degree vector $\mathbf{k}$ that are susceptible or infected at time t, and have partial degree vector $\mathbf{m}$. These quantities enumerate all possible node configurations over the course of any binary-state process. In other words, the sets $S_{\mathbf{k},\mathbf{m}}$ and $I_{\mathbf{k},\mathbf{m}}$ cannot be further partitioned, making $s_{\mathbf{k},\mathbf{m}}(t)$ and $i_{\mathbf{k},\mathbf{m}}(t)$ ideal functions of a rate equation formalism.

A dynamical process may be induced on such a network by assigning an initial state to each node, and allowing this state to evolve according to rates $F_{\mathbf{k},\mathbf{m}}$ and $R_{\mathbf{k},\mathbf{m}}$ per infinitesimal time step dt. The former is the rate of infection of susceptible nodes in $S_{\mathbf{k},\mathbf{m}}$ over a time interval dt, the latter the rate of recovery from the infected state for nodes in $I_{\mathbf{k},\mathbf{m}}$ over dt. This means all nodes sharing a degree vector $\mathbf{k}$ and partial degree vector $\mathbf{m}$ are equivalent in their rates of infection and recovery. The rate equations governing the sets $S_{\mathbf{k},\mathbf{m}}$ and $I_{\mathbf{k},\mathbf{m}}$ in a dynamics allowing both infection and recovery are

$$\begin{aligned}\frac{d}{dt}s_{\mathbf{k},\mathbf{m}} = -F_{\mathbf{k},\mathbf{m}}s_{\mathbf{k},\mathbf{m}} - \sum_{j=1}^{n}\beta_j^s(k_j - m_j)s_{\mathbf{k},\mathbf{m}} + \sum_{j=1}^{n}\beta_j^s(k_j - m_j + 1)s_{\mathbf{k},\mathbf{m}-\mathbf{e}_j} \\ + R_{\mathbf{k},\mathbf{m}}i_{\mathbf{k},\mathbf{m}} - \sum_{j=1}^{n}\gamma_j^s m_j s_{\mathbf{k},\mathbf{m}} + \sum_{j=1}^{n}\gamma_j^s(m_j+1)s_{\mathbf{k},\mathbf{m}+\mathbf{e}_j}\end{aligned} \tag{S1}$$

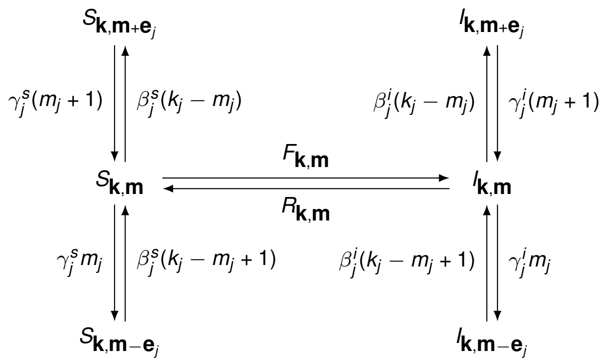

Figure S1: Representation of the AME system in Eqs. (S1)-(S2). The system constitutes an initial value problem, solved over a set of rate equations in $s_{\mathbf{k},\mathbf{m}}$ and $i_{\mathbf{k},\mathbf{m}}$, whose gain and loss terms, with their associated rates, are illustrated in the figure. The index $1 \leq j \leq n$ enumerates the gain or loss type, corresponding to the $4n+2$ ways in which a node may enter and leave class $S_{\mathbf{k},\mathbf{m}}$ or $I_{\mathbf{k},\mathbf{m}}$. This is possible through the infection and recovery of a node's neighbours (vertical movements) and through infection and recovery of a node itself (horizontal movements).

and

$$\begin{aligned}\frac{d}{dt}i_{\mathbf{k},\mathbf{m}} = F_{\mathbf{k},\mathbf{m}}s_{\mathbf{k},\mathbf{m}} - \sum_{j=1}^{n}\beta_j^i(k_j-m_j)i_{\mathbf{k},\mathbf{m}} + \sum_{j=1}^{n}\beta_j^i(k_j-m_j+1)i_{\mathbf{k},\mathbf{m}-\mathbf{e}_j} \\ - R_{\mathbf{k},\mathbf{m}}i_{\mathbf{k},\mathbf{m}} - \sum_{j=1}^{n}\gamma_j^i m_j i_{\mathbf{k},\mathbf{m}} + \sum_{j=1}^{n}\gamma_j^i(m_j+1)i_{\mathbf{k},\mathbf{m}+\mathbf{e}_j},\end{aligned} \tag{S2}$$

where $\mathbf{e}_j$ is the j-th basis vector of dimension n, and β_j^s, β_j^i, γ_j^s and γ_j^i the probabilities of a j-type neighbour becoming infected or recovering over an interval dt, calculated using the full system of $s_{\mathbf{k},\mathbf{m}}$ and $i_{\mathbf{k},\mathbf{m}}$ values. Explicitly, the β_j terms quantify the rate of infection of j-type neighbours for both susceptible and infected nodes,

$$\beta_j^s(t) = \frac{\sum_{k,\mathbf{k},\mathbf{m}} P(k)P(\mathbf{k})(k_j-m_j)F_{\mathbf{k},\mathbf{m}}s_{\mathbf{k},\mathbf{m}}(t)}{\sum_{k,\mathbf{k},\mathbf{m}} P(k)P(\mathbf{k})(k_j-m_j)s_{\mathbf{k},\mathbf{m}}(t)} \tag{S3a}$$

and

$$\beta_j^i(t) = \frac{\sum_{k,\mathbf{k},\mathbf{m}} P(k)P(\mathbf{k})(k_j-m_j)F_{\mathbf{k},\mathbf{m}}i_{\mathbf{k},\mathbf{m}}(t)}{\sum_{k,\mathbf{k},\mathbf{m}} P(k)P(\mathbf{k})(k_j-m_j)i_{\mathbf{k},\mathbf{m}}(t)}, \tag{S3b}$$

while the γ_j terms give the rate of recovery of j-type neighbours for both susceptible and infected nodes,

$$\gamma_j^s(t) = \frac{\sum_{k,\mathbf{k},\mathbf{m}} P(k)P(\mathbf{k})m_j R_{\mathbf{k},\mathbf{m}}s_{\mathbf{k},\mathbf{m}}(t)}{\sum_{k,\mathbf{k},\mathbf{m}} P(k)P(\mathbf{k})m_j s_{\mathbf{k},\mathbf{m}}(t)}, \tag{S4a}$$

and

$$\gamma_j^i(t) = \frac{\sum_{k,\mathbf{k},\mathbf{m}} P(k)P(\mathbf{k})m_j R_{\mathbf{k},\mathbf{m}}i_{\mathbf{k},\mathbf{m}}(t)}{\sum_{k,\mathbf{k},\mathbf{m}} P(k)P(\mathbf{k})m_j i_{\mathbf{k},\mathbf{m}}(t)}, \tag{S4b}$$

where we sum over $k_{min} \leq k \leq k_{max}$, all $\mathbf{k}$ such that $\sum_j k_j = k$, and all $\mathbf{m}$ such that $0 \leq m_j \leq k_j$. The values $s_{\mathbf{k},\mathbf{m}}(t)$ and $i_{\mathbf{k},\mathbf{m}}(t)$ combined with the degree and degree vector distributions $P(k)$ and $P(\mathbf{k})$ give us the density of infected nodes $\rho(t)$,

$$\rho(t) = 1 - \sum_{k,\mathbf{k},\mathbf{m}} P(k)P(\mathbf{k})s_{\mathbf{k},\mathbf{m}}(t) = \sum_{k,\mathbf{k},\mathbf{m}} P(k)P(\mathbf{k})i_{\mathbf{k},\mathbf{m}}(t). \tag{S5}$$

The initial conditions are prescribed by $i_{\mathbf{k},\mathbf{m}}(0)$ and $s_{\mathbf{k},\mathbf{m}}(0)$, subject to the normalisation condition

$$\sum_{\mathbf{m}} i_{\mathbf{k},\mathbf{m}}(t) + \sum_{\mathbf{m}} s_{\mathbf{k},\mathbf{m}}(t) = 1. \tag{S6}$$

As such, we have defined a closed system of deterministic equations that can be solved numerically using standard methods (Fig. S1).

S1.2 Monotone dynamics

The above derivation assumes generic infection and recovery rates $F_{\mathbf{k},\mathbf{m}}$ and $R_{\mathbf{k},\mathbf{m}}$. In this section, we illustrate a solution of the AMEs particular to monotone dynamics with the example of a threshold rule for complex contagion. This may be generalized to other monotone or non-recovery dynamics, where $R_{\mathbf{k},\mathbf{m}} = 0$. In dynamical processes on weighted networks, we are typically interested in the node properties relating to the edge weight. We define the strength of a node as the sum of edge weights across all neighbours, $q_{\mathbf{k}} = \mathbf{k}\cdot\mathbf{w}$. Similarly, we define the partial strength as the sum of edge weights across all infected neighbours, $q_{\mathbf{m}} = \mathbf{m}\cdot\mathbf{w}$, with $0 \leq q_{\mathbf{m}} \leq q_{\mathbf{k}}$. The infection rate for complex contagion can thus be expressed as

$$F_{\mathbf{k},\mathbf{m}} = \begin{cases} p & q_{\mathbf{m}} < \phi q_{\mathbf{k}} \\ 1 & q_{\mathbf{m}} \geq \phi q_{\mathbf{k}} \end{cases}, \quad k > 0, \tag{S7}$$

with rate of recovery $R_{\mathbf{k},\mathbf{m}} = 0$. Here, p is the rate of spontaneous infection, whereby a susceptible node may become infected independently of the state of its neighbours. The threshold ϕ is the fraction of a node's

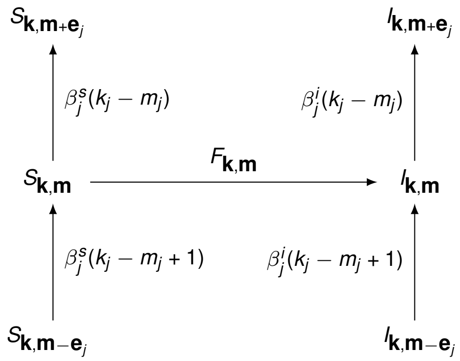

Figure S2: Representation of the AMEs for monotone dynamics. A recovery rate $R_{\mathbf{k},\mathbf{m}} = 0$ implies $\gamma_j^i = \gamma_j^s = 0$, so the rate equations for $S_{\mathbf{k},\mathbf{m}}$ and $I_{\mathbf{k},\mathbf{m}}$ are characterized by only $2n + 1$ gain and loss terms, in contrast to Fig. S1.

total strength that must be met by the partial strength for that node to undergo induced infection. In other words, it is the fraction of a node's total received influence that must come from infected neighbours before that node itself becomes infected. Correspondingly, the master equations become (Fig. S2)

$$\frac{d}{dt}s_{\mathbf{k},\mathbf{m}} = -F_{\mathbf{k},\mathbf{m}}s_{\mathbf{k},\mathbf{m}} - \sum_{j=1}^{n}\left(\beta_j^s(k_j-m_j)s_{\mathbf{k},\mathbf{m}} - \beta_j^s(k_j-m_j+1)s_{\mathbf{k},\mathbf{m}-\mathbf{e}_j}\right) \tag{S8a}$$

$$\frac{d}{dt}i_{\mathbf{k},\mathbf{m}} = +F_{\mathbf{k},\mathbf{m}}s_{\mathbf{k},\mathbf{m}} - \sum_{j=1}^{n}\left(\beta_j^i(k_j-m_j)i_{\mathbf{k},\mathbf{m}} - \beta_j^i(k_j-m_j+1)i_{\mathbf{k},\mathbf{m}-\mathbf{e}_j}\right). \tag{S8b}$$

The AME system (S8) is decoupled, so we may only consider the equation for $s_{\mathbf{k},\mathbf{m}}$ when, for example, reducing the AMEs to a lower-dimensional system.

S1.3 Monotone dynamics for bimodal weight distribution

Here we analyse the simple case of a network with arbitrary degree distribution $P(k)$ and $n = 2$ edge weights, $w_1, w_2 > 0$, which may or may not appear with equal probability in the network. The probability distribution $P(w)$ of a randomly chosen weight w is

$$P(w) = \begin{cases} \delta & w = w_1 \\ 1-\delta & w = w_2 \end{cases}, \tag{S9}$$

and 0 elsewhere, with $\delta \in (0,1)$. Assuming $w_1 \geq w_2$ for the sake of simplicity, δ is the fraction of strong edges in the network, and thus contributes to skewness in the weight distribution. The weight average and standard deviation are given by

$$\mu = \delta w_1 + (1-\delta)w_2 \quad \text{and} \quad \sigma = \sqrt{\sum\nolimits_w (w-\mu)^2 P(w)} = \sqrt{\delta(1-\delta)}(w_1 - w_2). \tag{S10}$$

We may invert the linear system in Eq. (S10) to obtain the strong and weak weights, w_1 and w_2, in terms of μ and σ,

$$w_1 = \mu + \sqrt{\frac{1-\delta}{\delta}}\sigma \tag{S11}$$

$$w_2 = \mu - \sqrt{\frac{\delta}{1-\delta}}\sigma, \tag{S12}$$

where $\sigma \geq 0$ and $\mu > \sigma\sqrt{\delta/(1-\delta)}$ [1]. Thus, we can take μ and σ as parameters, and use Eq. (S11) to obtain values for the weights in the network.

As for the AME formalism in the case of a bimodal weight distribution, the weight, degree and partial degree vectors are $\mathbf{w} = (w_1, w_2)^{\mathrm{T}}$, $\mathbf{k} = (k_1, k_2)^{\mathrm{T}}$, and $\mathbf{m} = (m_1, m_2)^{\mathrm{T}}$, respectively, subject to the constraints $k = k_1 + k_2$ and $m = m_1 + m_2$. Moreover, $q_{\mathbf{k}} = k_1 w_1 + k_2 w_2$ and $q_{\mathbf{m}} = m_1 w_1 + m_2 w_2$. Due to the

[1] This second condition is necessary to have positive weights only, but is not required by the following results.

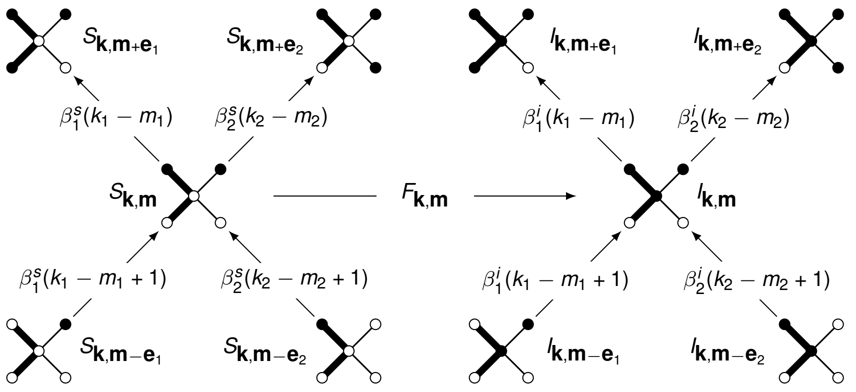

Figure S3: Representation of gain and loss sets for node class $\mathbf{k} = (2, 2)$, $\mathbf{m} = (1, 1)$. Nodes in sets $S_{\mathbf{k},\mathbf{m}}$ and $I_{\mathbf{k},\mathbf{m}}$ have two possible ways of entering and exiting the class through neighbour infection. This figure corresponds to the set of $\mathbf{k} = (2, 2)$ nodes in Fig. S4.

degree constraint and Eq. (S9), the degree vector takes the values $\mathbf{k} = (0, k), (1, k - 1), \ldots, (k, 0)$, which are binomially distributed in the network according to

$$P(\mathbf{k}) = \binom{k}{k_1} \delta^{k_1} (1 - \delta)^{k-k_1} = B_{k,k_1}(\delta), \tag{S13}$$

Further, the sum over degrees, degree vectors and partial degree vectors can be written explicitly as

$$\sum_{k,\mathbf{k},\mathbf{m}} = \sum_{k=k_{min}}^{k_{max}} \sum_{k_1=0}^{k} \sum_{m_1=0}^{k_1} \sum_{m_2=0}^{k_2} . \tag{S14}$$

With Eqs. (S13)-(S14) and a given degree distribution $P(k)$, we may write explicitly the full and reduced AME systems, solve them numerically, and explore the behaviour of the fraction of infected nodes $\rho(t)$ as a function of all parameters.

The bimodal case is ideal as a means of illustrating how a given node may occupy a series of $(\mathbf{k}, \mathbf{m})$ classes over the course of a dynamical process. By taking the example of a node in class $\mathbf{k} = (2, 2)$, $\mathbf{m} = (1, 1)$ adhering to the infection rate $F_{\mathbf{k},\mathbf{m}}$ (Fig. S3), we illustrate the interdependencies of various node classes and possible flows between them. This class corresponds to nodes with degree $k = 4$, consisting of two strong and two weak neighbours, one of each being infected. It follows that two ways in which a node may leave this class is by an additional neighbour of either type becoming infected. Similarly, two ways in which a node may enter the class is by having only one infected neighbour of either edge type, and gaining an infected neighbour of the opposite type (Fig. S4). We note that although the degree vector $\mathbf{k}$ of a node is fixed throughout the dynamical process, its partial degree vector $\mathbf{m}$ is free to change according to the number and edge-types of its infected neighbours. This allows us to attribute relative sizes to each of the $(\mathbf{k}, \mathbf{m})$ classes. A node's class is a dynamic quantity, and it is the flow of nodes through each class that we use to characterize the state of the system through $s_{\mathbf{k},\mathbf{m}}(t)$ and $i_{\mathbf{k},\mathbf{m}}(t)$ over time.

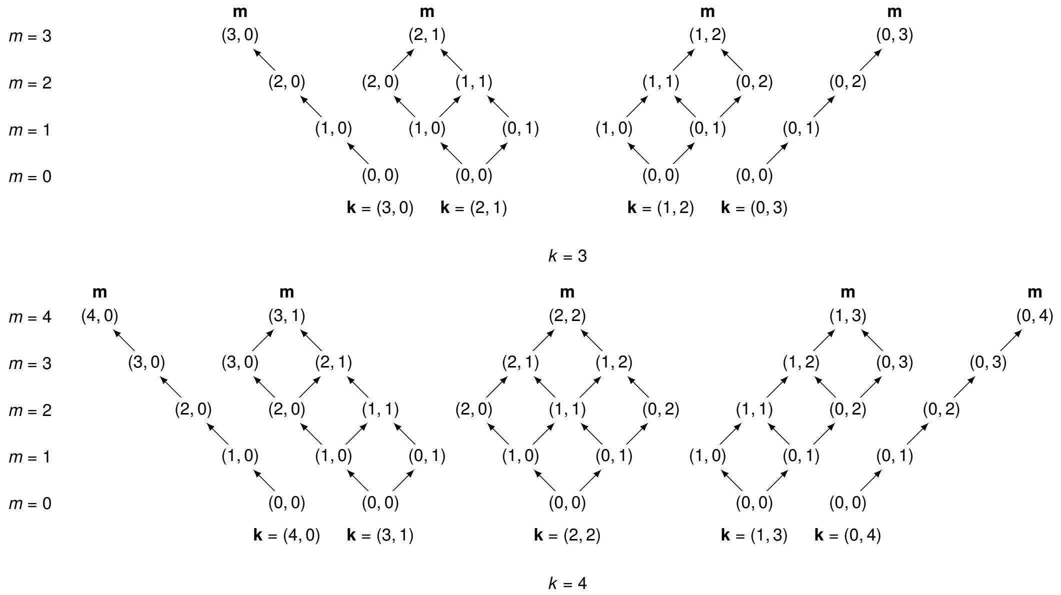

Figure S4: Possible $(\mathbf{k}, \mathbf{m})$ classes for $k = 3, 4$ with $n = 2$, and flows between them. Note that it is impossible for a node to move between classes of different $\mathbf{k}$, since the degree vector of a node is fixed in time. In the non-recovery model of the figure, it is also impossible to make a downward transition to a class with lower m.

S1.4 Reduced AMEs

To reduce the dimension of the weighted AMEs for monotone dynamics [Eq. (S8)] in the case of a stepwise infection rate $F_{\mathbf{k},\mathbf{m}}$ [Eq. (S7)], we need to consider system-wide quantities that are more aggregated than $s_{\mathbf{k},\mathbf{m}}$. We take the probability $\rho(t)$ that a randomly chosen node is infected, i.e. the fraction of infected nodes in the network, and the probability $\nu_j(t)$ that a randomly chosen neighbour (across a j-type edge) of a susceptible node is infected (see Methods). We start by proposing an exact solution for the AME system in terms of the ansatz

$$s_{\mathbf{k},\mathbf{m}}(t) = e^{-pt} \prod_{j=1}^{n} B_{k_j,m_j}[\nu_j(t)] \quad \text{for} \quad q_{\mathbf{m}} < \phi q_{\mathbf{k}}, \tag{S15}$$

where $B_{k_j,m_j} = \binom{k_j}{m_j} \rho^{m_j} (1-\rho)^{k_j - m_j}$ is the binomial distribution. The meaning of the ansatz in Eq. (S15) is quite intuitive and takes into account two processes. First, a susceptible node with k_j edges of type j, is connected to m_j infected nodes with the binomially distributed probability $B_{k_j,m_j}(\nu_j)$. Second, for $q_{\mathbf{m}} < \phi q_{\mathbf{k}}$ a susceptible node does not fulfill the threshold rule and can only become infected spontaneously with probability e^{-pt}, since the system is progressively being filled due to spontaneous infection. Considering these processes as independent leads to the product in Eq. (S15).

The next step is to insert the ansatz (S15) into the AME system (S8) and derive a set of ordinary differential equations (ODEs) for the aggregated quantities ρ and ν_j. Taking the time derivative $\dot{s}_{\mathbf{k},\mathbf{m}}$ of

Eq. (S15) (i.e. the left-hand side of the AME system) we get

$$\dot{s}_{\mathbf{k},\mathbf{m}} = \left(\sum_{j=1}^{n} \left[\frac{m_j}{\nu_j} - \frac{k_j - m_j}{1-\nu_j} \right] \dot{\nu}_j - p \right) s_{\mathbf{k},\mathbf{m}}. \tag{S16}$$

Then, we use the infection rate of weighted contagion for $q_{\mathbf{m}} < \phi q_{\mathbf{k}}$, the ansatz (S15) and the binomial identity

$$B_{k_j,m_j-1}(\nu_j) = \frac{1-\nu_j}{\nu_j} \frac{m_j}{k_j - m_j + 1} B_{k_j,m_j}(\nu_j), \tag{S17}$$

in the right-hand side of the AME system to obtain

$$-F_{\mathbf{k},\mathbf{m}} s_{\mathbf{k},\mathbf{m}} - \sum_{j=1}^{n} \beta_j^s (k_j - m_j) s_{\mathbf{k},\mathbf{m}} + \sum_{j=1}^{n} \beta_j^s (k_j - m_j + 1) s_{\mathbf{k},\mathbf{m}-\mathbf{e}_j} = \left[-p + \sum_{j=1}^{n} \beta_j^s \left(m_j - k_j + \frac{1-\nu_j}{\nu_j} m_j \right) \right] s_{\mathbf{k},\mathbf{m}}. \tag{S18}$$

Equating Eqs. (S16)-(S18) as in the AME system, and separating terms for a given value of j from the rest ($i \neq j$) leads to

$$\frac{(1-\nu_j)m_j + \nu_j(m_j - k_j)}{\nu_j} \left(\frac{\dot{\nu}_j}{1-\nu_j} - \beta_j^s \right) = \sum_{i \neq j}^{n} \frac{(1-\nu_i)m_i + \nu_i(m_i - k_i)}{\nu_i} \left(\beta_i^s - \frac{\dot{\nu}_i}{1-\nu_i} \right). \tag{S19}$$

Since the left-hand side of Eq. (S19) depends on the function ν_j and its derivative only, while the right-hand side depends on the rest of the functions ν_i, both sides must be equal to some constant c_j. For the left-hand side, this means that

$$\frac{\dot{\nu}_j}{1-\nu_j} - \beta_j^s = c_j \frac{\nu_j}{m_j - \nu_j k_j}, \quad \forall m_j, k_j. \tag{S20}$$

For the ODE (S20) to hold regardless of the values of m_j and k_j, we need $c_j = 0$. Then, the condition on ν_j such that the ansatz (S15) is a solution of the AME system is

$$\frac{\dot{\nu}_j}{1-\nu_j} = \beta_j^s. \tag{S21}$$

This ODE has the initial condition $\nu_j(0) = \rho(0) = 0$, obtained by evaluating Eq. (S15) at $t = 0$ and comparing with the expression $B_{k_j,m_j}(0)$, which corresponds to an infinitesimally small initial infection randomly distributed in the network (see Methods).

The next step is to extend a general result derived by Gleeson in [2] [Eqs. (F6)–(F10) therein] to the case of weighted networks. We start by multiplying the AME system (S8) by $P(k)P(\mathbf{k})(k_j - m_j)$ and summing

over k, $\mathbf{k}$, and $\mathbf{m}$,

$$\frac{d}{dt}\sum_{k,\mathbf{k},\mathbf{m}} P(k)P(\mathbf{k})(k_j - m_j)s_{\mathbf{k},\mathbf{m}} = -\sum_{k,\mathbf{k},\mathbf{m}} P(k)P(\mathbf{k})(k_j - m_j)F_{\mathbf{k},\mathbf{m}}s_{\mathbf{k},\mathbf{m}}$$
$$-\sum_{k,\mathbf{k},\mathbf{m}} P(k)P(\mathbf{k})\sum_{i=1}^{n}\beta_i^s(k_j - m_j)\big[(k_i - m_i)s_{\mathbf{k},\mathbf{m}} - (k_i - m_i + 1)s_{\mathbf{k},\mathbf{m}-\mathbf{e}_i}\big]. \tag{S22}$$

From the definition of β_j^s in Eq. (S3), the first term on the right hand side of Eq. (S22) may be written as

$$-\beta_j^s\sum_{k,\mathbf{k},\mathbf{m}} P(k)P(\mathbf{k})(k_j - m_j)s_{\mathbf{k},\mathbf{m}}. \tag{S23}$$

As for the second term on the right hand side, when $i = j$ the term telescopes to Eq. (S23), and for $i \neq j$ it telescopes to 0. Overall, we can rearrange Eq. (S22) and obtain

$$\beta_j^s = -\frac{1}{2}\frac{d}{dt}\ln\sum_{k,\mathbf{k},\mathbf{m}} P(k)P(\mathbf{k})(k_j - m_j)s_{\mathbf{k},\mathbf{m}}. \tag{S24}$$

Since $\beta_j^s = -\frac{d}{dt}\ln(1-\nu_j)$ from Eq. (S21), equating Eqs. (S21)-(S24) implies that

$$d_j(1-\nu_j)^2 = \sum_{k,\mathbf{k},\mathbf{m}} P(k)P(\mathbf{k})(k_j - m_j)s_{\mathbf{k},\mathbf{m}}, \tag{S25}$$

with d_j a constant that can be determined from initial conditions. Assuming an infinitesimally small fraction of infected nodes randomly distributed in the network (see Methods), and since $\nu_j(0) = \rho(0) = 0$ and $B_{k_i,m_i}(0) = \delta_{m_i,0}$ with δ_{ij} the Kronecker delta, we have

$$d_j = \sum_{k,\mathbf{k},\mathbf{m}} P(k)P(\mathbf{k})(k_j - m_j)\prod_{i=1}^{n} B_{k_i,m_i}(0) = \sum_{k,\mathbf{k}} P(k)P(\mathbf{k})k_j = z_j, \tag{S26}$$

where z_j is the average number of j-type edges a node has in the network, or average j-degree. Thus,

$$\sum_{k,\mathbf{k},\mathbf{m}} P(k)P(\mathbf{k})(k_j - m_j)s_{\mathbf{k},\mathbf{m}} = z_j(1-\nu_j)^2. \tag{S27}$$

The next step is to use Eq. (S27) to find a new expression for β_j^s and thus write the ODE (S21) explicitly in terms of ν_j. Noting that the left-hand side of Eq. (S27) is the denominator in the definition of β_j^s, we get

$$\begin{aligned}
\beta_j^s &= \frac{1}{z_j(1-\nu_j)^2}\left[p\sum_{k,\mathbf{k}} P(k)P(\mathbf{k})\sum_{q_\mathbf{m}<\phi q_\mathbf{k}}(k_j - m_j)s_{\mathbf{k},\mathbf{m}} + \sum_{k,\mathbf{k}} P(k)P(\mathbf{k})\sum_{q_\mathbf{m}\geq\phi q_\mathbf{k}}(k_j - m_j)s_{\mathbf{k},\mathbf{m}}\right] \\
&= \frac{1}{z_j(1-\nu_j)^2}\left[z_j(1-\nu_j)^2 - (1-p)\sum_{k,\mathbf{k}} P(k)P(\mathbf{k})\sum_{q_\mathbf{m}<\phi q_\mathbf{k}}(k_j - m_j)s_{\mathbf{k},\mathbf{m}}\right] \\
&= \frac{1}{1-\nu_j}\left[1 - \nu_j - (1-p)e^{-pt}\sum_{k,\mathbf{k}}\frac{k_j}{z_j}P(k)P(\mathbf{k})\sum_{q_\mathbf{m}<\phi q_\mathbf{k}} B_{k_j-1,m_j}(\nu_j)\prod_{i\neq j}^{n} B_{k_i,m_i}(\nu_i)\right],
\end{aligned} \tag{S28}$$

where the sums $\sum_{q_{\mathbf{m}}<\phi q_{\mathbf{k}}}$ and $\sum_{q_{\mathbf{m}}\geq\phi q_{\mathbf{k}}}$ run over all partial degree vectors $\mathbf{m}$ that satisfy their respective inequalities, and we have also inserted the ansatz (S15) and the binomial identity $(k_j - m_j)B_{k_j,m_j}(\nu_j) = k_j(1-\nu_j)B_{k_j-1,m_j}(\nu_j)$ to simplify the expression of β_j^s. Moreover, we may introduce the *response function* of the monotone, threshold-driven dynamics of our model,

$$f(\mathbf{k},\mathbf{m}) = \begin{cases} 0 & q_{\mathbf{m}} < \phi q_{\mathbf{k}} \\ 1 & q_{\mathbf{m}} \geq \phi q_{\mathbf{k}} \end{cases}, \quad k > 0, \tag{S29}$$

with $f(\mathbf{0},\mathbf{0}) = 0$ (a function that activates when a $(\mathbf{k},\mathbf{m})$-class node fulfils the threshold condition and gets infected), in order to invert the restricted sum of Eq. (S28),

$$\begin{aligned}\sum_{q_{\mathbf{m}}<\phi q_{\mathbf{k}}} B_{k_j-1,m_j}(\nu_j)\prod_{i\neq j}^{n} B_{k_i,m_i}(\nu_i) &= \sum_{\mathbf{m}}[1-f(\mathbf{k},\mathbf{m})]B_{k_j-1,m_j}(\nu_j)\prod_{i\neq j}^{n} B_{k_i,m_i}(\nu_i) \\ &= 1 - \sum_{q_{\mathbf{m}}\geq\phi q_{\mathbf{k}}} B_{k_j-1,m_j}(\nu_j)\prod_{i\neq j}^{n} B_{k_i,m_i}(\nu_i).\end{aligned} \tag{S30}$$

Overall, comparing Eqs. (S21)-(S28), we can write an explicit ODE for ν_j,

$$\frac{d}{dt}\nu_j = g_j(\boldsymbol{\nu},t) - \nu_j, \tag{S31}$$

with $\boldsymbol{\nu} = (\nu_1,\ldots,\nu_n)^{\mathrm{T}}$, $j = 1,\ldots,n$, and the function $g_j(\boldsymbol{\nu},t)$ given by

$$g_j(\boldsymbol{\nu},t) = f_t + (1-f_t)\sum_{k,\mathbf{k}}\frac{k_j}{z_j}P(k)P(\mathbf{k})\sum_{q_{\mathbf{m}}\geq\phi q_{\mathbf{k}}} B_{k_j-1,m_j}(\nu_j)\prod_{i\neq j}^{n} B_{k_i,m_i}(\nu_i), \tag{S32}$$

where we have defined $f_t = 1-(1-p)e^{-pt}$.

Even though Eq. (S31) is closed and in this sense equivalent to the AME system (S8), we may also derive a corresponding ODE for ρ, since we are mainly interested in the temporal evolution of the fraction of infected nodes in the network. From the definition of ρ and the AME system we have

$$\begin{aligned}\dot{\rho} = -\sum_{k,\mathbf{k},\mathbf{m}} P(k)P(\mathbf{k})\dot{s}_{\mathbf{k},\mathbf{m}} &= \sum_{k,\mathbf{k},\mathbf{m}} P(k)P(\mathbf{k})F_{\mathbf{k},\mathbf{m}}s_{\mathbf{k},\mathbf{m}} \\ &+ \sum_{k,\mathbf{k},\mathbf{m}} P(k)P(\mathbf{k})\sum_{j=1}^{n}\beta_j^s\left[(k_j-m_j)s_{\mathbf{k},\mathbf{m}} - (k_j-m_j+1)s_{\mathbf{k},\mathbf{m}-\mathbf{e}_j}\right],\end{aligned} \tag{S33}$$

where the second term in the right-hand side telescopes to zero. Then, we use an algebraic manipulation similar to that of Eq. (S28) to obtain

$$\begin{aligned}\dot{\rho} &= p\sum_{k,\mathbf{k}} P(k)P(\mathbf{k})\sum_{q_{\mathbf{m}}<\phi q_{\mathbf{k}}} s_{\mathbf{k},\mathbf{m}} + \sum_{k,\mathbf{k}} P(k)P(\mathbf{k})\sum_{q_{\mathbf{m}}\geq\phi q_{\mathbf{k}}} s_{\mathbf{k},\mathbf{m}} \\ &= 1-\rho-(1-p)e^{-pt}\sum_{k,\mathbf{k}} P(k)P(\mathbf{k})\sum_{q_{\mathbf{m}}<\phi q_{\mathbf{k}}}\prod_{j=1}^{n} B_{k_j,m_j}(\nu_j).\end{aligned} \tag{S34}$$

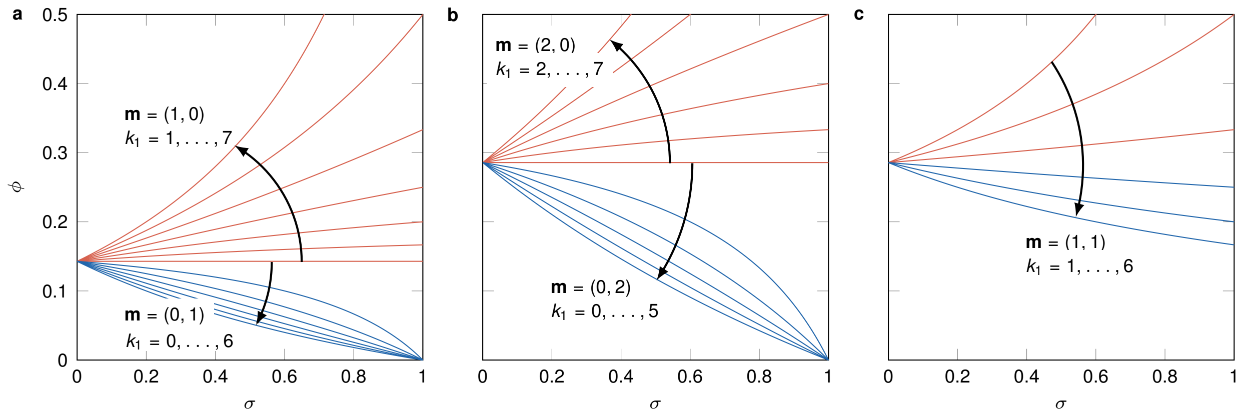

Figure S5: Phase boundaries in (σ, ϕ)-parameter space for a k-regular random network ($k = 7$) with $m = 1, 2$. (a) Boundaries of regions where just one infected neighbour of type $j = 1, 2$ is sufficient to induce infection. Curves in red indicate that the associated $(\mathbf{k}, \mathbf{m})$ class produces a speed-up effect on the spreading process relative to the same process on an unweighted network. Conversely, classes associated with the curves in blue produce a slow-down effect on cascades. (b) Similar boundaries for the networks where two infected neighbours of the same type are sufficient to cause induced infection, over a range of degree vectors. (c) Boundaries where one infected neighbour of each type causes infection.

Thus, the ODE for ρ is

$$\frac{d}{dt}\rho = h(\boldsymbol{\nu}, t) - \rho, \tag{S35}$$

where the function $h(\boldsymbol{\nu}, t)$ is given by

$$h(\boldsymbol{\nu}, t) = f_t + (1 - f_t) \sum_{k,\mathbf{k}} P(k) P(\mathbf{k}) \sum_{q_\mathbf{m} \geq \phi q_\mathbf{k}} \prod_{j=1}^{n} B_{k_j, m_j}(\nu_j). \tag{S36}$$

Combining all of these results, the AME system (S8) is reduced to a closed system of $n + 1$ coupled, non-linear ODEs,

$$\dot{\nu}_j = g_j(\boldsymbol{\nu}, t) - \nu_j, \tag{S37a}$$

$$\dot{\rho} = h(\boldsymbol{\nu}, t) - \rho, \tag{S37b}$$

with the quantities $g_j(\boldsymbol{\nu}, t)$ and $h(\boldsymbol{\nu}, t)$ given explicitly by Eqs. (S32)-(S36).

S2 Combinatorial solution of parameter space boundaries

The dynamics of threshold driven contagion on weighted networks depends on the stepwise infection rate $F_{\mathbf{k},\mathbf{m}}$ of Eq. (S7). Considering the case of equality, $q_\mathbf{m} = \phi q_\mathbf{k}$, and writing $q_\mathbf{k}$ and $q_\mathbf{m}$ explicitly, we obtain $\phi = \mathbf{m}\cdot\mathbf{w}/\mathbf{k}\cdot\mathbf{w}$. Noting that the σ dependence is contained in the weight vector $\mathbf{w}$, we solve for $\mathbf{k}$ and $\mathbf{m}$, and associate the solution with a unique boundary in (σ, ϕ)-parameter space separating regions of differing t_r,

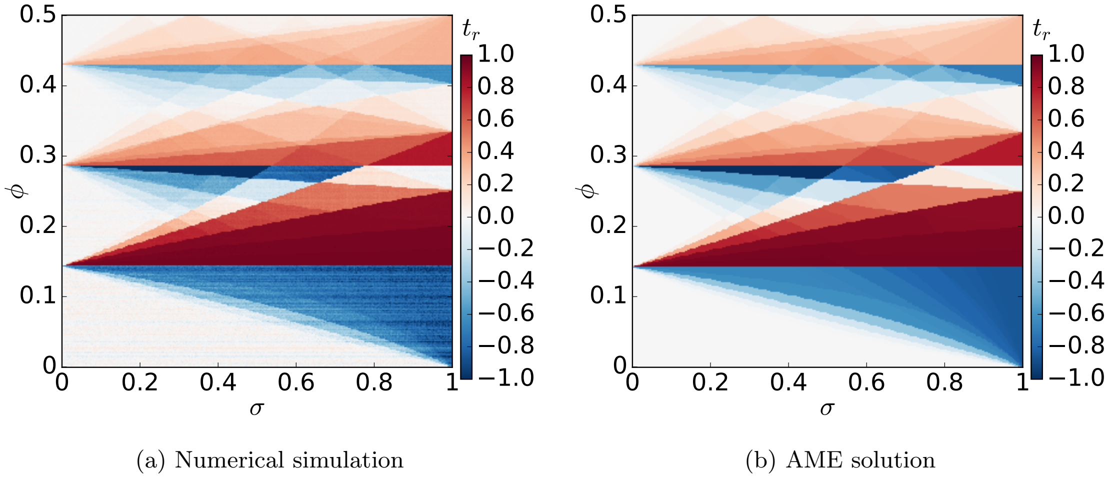

Figure S6: Comparison between numerical simulations and AME solutions. (σ, ϕ)-parameter space for the relative time t_r of cascade emergence, obtained by Monte Carlo numerical simulations (a) and the numerical solutions of the full and reduced AME systems (b), the last two of which are indistinguishable. Numerical simulations consider k-regular random networks ($k = 7$) with $N = 10^4$, $p = 2 \times 10^{-4}$, and averages over 25 realisations.

the relative time of cascade emergence (Fig. S5). In other words, boundaries for t_r in (σ, ϕ)-parameter space separate network configurations where the corresponding $(\mathbf{k}, \mathbf{m})$ class does and does not satisfy the threshold rule $q_{\mathbf{m}} \geq \phi q_{\mathbf{k}}$, thus promoting or hindering spreading. In Fig. S5 we enumerate all possible boundaries for up to two infected neighbours in the case of a k-regular random network ($k = 7$) and a bimodal weight distribution ($n = 2$). Fig. S5a shows the case where one strong infected neighbour, $\mathbf{m} = (1, 0)$, is sufficient to cause infection for nodes with $k_1 = 1, \dots, k$ strong neighbours. These curves are shown in red, since the corresponding node classes induce a faster cascade of spreading compared to the same process carried out on an unweighted network. Since the weight vector is $\mathbf{w} = (\mu + \sigma, \mu - \sigma)^T$ for weight mean μ and skewness $\delta = 0.5$, boundaries can be written explicitly as

$$\phi = \frac{m_1 w_1 + m_2 w_2}{k_1 w_1 + k_2 w_2} = \frac{\mu(m_1 + m_2) + \sigma(m_1 - m_2)}{\mu(k_1 + k_2) + \sigma(k_1 - k_2)}. \tag{S38}$$

Curves in blue are the boundaries where one weak infected neighbour, $\mathbf{m} = (0, 1)$, is sufficient to induce infection (and a slower cascade than the unweighted case). These curves are enumerated by the number of strong neighbours, $k_1 = 0, \dots, k - 1$. Curves in Fig. S5b are analogous to Fig. S5a, except replacing $\mathbf{m} = (1, 0)$ and $\mathbf{m} = (0, 1)$ with $\mathbf{m} = (2, 0)$ and $\mathbf{m} = (0, 2)$. Finally, Fig. S5c corresponds to the boundaries due to node sets with $\mathbf{m} = (1, 1)$, where having two infected neighbours, one of each type, is sufficient for these classes to undergo induced infection.

S3 Comparison of numerical experiment and AME solutions

As discussed in the main text, the behaviour of threshold driven contagion over weighted networks, in Monte Carlo simulations or as predicted by numerically computing AME solutions, is remarkably consistent. We

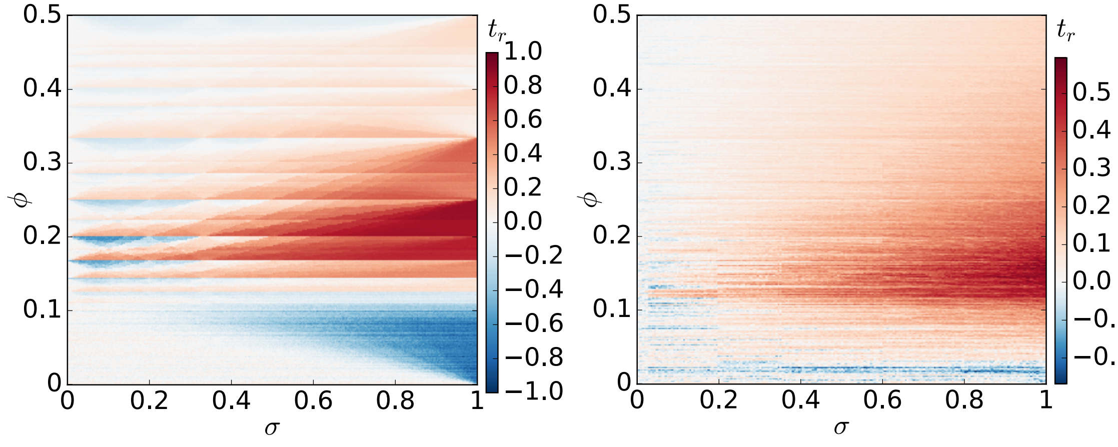

(a) Random network

(b) Pardus social network

Figure S7: Other heterogeneous synthetic and real networks. (σ, ϕ)-parameter space for the relative time t_r of cascade emergence, simulated on a configuration-model random network (a) and the Pardus signed social network (b). Numerical simulations on (a) consider networks with $N = 10^4$, average degree $z = 7$ and averages over 25 realisations. All simulations correspond to $p = 2 \times 10^{-4}$.

have illustrated this similarity by comparing t_r values over (σ, ϕ)-parameter space, as well as by plotting the temporal evolution of the infection density ρ and other quantities characterizing the dynamics. To further compare the (σ, ϕ)-parameter space between Monte Carlo simulations and AME solutions, we show them side-by-side in Fig. S6 and quantify their similarity by computing the mean absolute difference

$$MD[t_r(\sigma, \phi)] = \frac{\sum_\sigma \sum_\phi |t_r^{\text{sim}}(\sigma, \phi) - t_r^{\text{theo}}(\sigma, \phi)|}{N_\sigma N_\phi}, \tag{S39}$$

where N_σ and N_ϕ are the number of points considered in each dimension of the parameter space, and $t_r^{\text{sim}}(\sigma, \phi)$ and $t_r^{\text{theo}}(\sigma, \phi)$ are the relative times of cascade emergence for a given (σ, ϕ) point, measured by numerical simulations or AME solutions. This quantity is very small, $MD = 2.8 \times 10^{-7}$, which indicates that despite making several simplifying assumptions during the derivation of the full and reduced AME systems [Eqs. (S8)-(S37)], they provide an extremely good approximation of the spreading process with differences only due to small statistical fluctuations in finite-size numerical simulations.

S4 Other heterogeneous synthetic and real networks

In the main text we have seen that, even for heterogeneous synthetic and real world networks, threshold driven contagion strongly depends on link weights via simple mechanisms that can be understood by master equations or combinatorial arguments, and develops spreading cascades that are either faster of slower than their counterparts in unweighted contagion, depending on the values of σ and ϕ. Here we further support this argument by exploring two additional examples of synthetic and empirical networks. The synthetic structure is a configuration-model random network with average degree $z = 7$, and bimodal weight distribution with average $\mu = 1$ and skewness $\delta = 0.5$ (Fig. S7a). The (σ, ϕ)-parameter space for t_r is qualitatively very

similar to the ones observed for configuration-model k-regular or scale-free networks, with several fast and slow cascade regimes that start from values on the ϕ axis determined by the harmonic series of degrees present in the network. The empirical structure is a signed social network, the alliance / enemy network of the Pardus massive multiplayer online game [5]. This network consists of $N = 4650$ nodes connected by $66,580$ links, of which a fraction $\delta = 0.64$ have a positive alliance sign (and are considered as strong ties by us), while the rest of the links have a negative enemy sign and are interpreted as weak. The (σ, ϕ)-parameter space for t_r (Fig. S7b) is somewhat less structured than in the other explored networks, but still shows regions of fast and slow cascades with respect to the unweighted case.

References

[1] Porter, M. A. & Gleeson, J. P. Dynamical systems on networks. *Frontiers in Applied Dynamical Systems: Reviews and Tutorials* **4** (2016).

[2] Gleeson, J. P. Binary-state dynamics on complex networks: Pair approximation and beyond. *Phys. Rev. X* **3**, 021004 (2013).

[3] Gleeson, J. P. Cascades on correlated and modular random networks. *Phys. Rev. E* **77**, 046117 (2008).

[4] Gleeson, J. P. High-accuracy approximation of binary-state dynamics on networks. *Phys. Rev. Lett.* **107**, 068701 (2011).

[5] Szell, M., Lambiotte, R. & Thurner, S. Multirelational organization of large-scale social networks in an online world. *Proc. Natl. Acad. Sci. U.S.A.* **107**, 13636–13641 (2010).
